# Temporal and spatial cellular and molecular pathological alterations with single-cell resolution in the adult spinal cord after injury

**DOI:** 10.1038/s41392-022-00885-4

**Published:** 2022-03-02

**Authors:** Chen Li, Zhourui Wu, Liqiang Zhou, Jingliang Shao, Xiao Hu, Wei Xu, Yilong Ren, Xingfei Zhu, Weihong Ge, Kunshan Zhang, Jiping Liu, Runzhi Huang, Jing Yu, Dandan Luo, Xuejiao Yang, Wenmin Zhu, Rongrong Zhu, Changhong Zheng, Yi Eve Sun, Liming Cheng

**Affiliations:** 1 Division of Spine, Department of Orthopaedics, Tongji Hospital, Tongji University School of Medicine, Shanghai, 200065 China; 2 Key Laboratory of Spine and Spinal cord Injury Repair and Regeneration (Tongji University), Ministry of Education, Shanghai, 200072 China; 3 Institute of Spinal and Spinal Cord Injury, Tongji University School of Medicine, Shanghai, 200065 China; 4 Stem Cell Translational Research Center, Tongji Hospital, Tongji University School of Medicine, Shanghai, 200065 China; 5 Department of Psychiatry and Biobehavioral Sciences, David Geffen School of Medicine, University of California Los Angeles, Los Angeles, CA 90095 USA

**Keywords:** Cellular neuroscience, Diseases of the nervous system, Regeneration and repair in the nervous system

## Abstract

Spinal cord injury (SCI) involves diverse injury responses in different cell types in a temporally and spatially specific manner. Here, using single-cell transcriptomic analyses combined with classic anatomical, behavioral, electrophysiological analyses, we report, with single-cell resolution, temporal molecular and cellular changes in crush-injured adult mouse spinal cord. Data revealed pathological changes of 12 different major cell types, three of which infiltrated into the spinal cord at distinct times post-injury. We discovered novel microglia and astrocyte subtypes in the uninjured spinal cord, and their dynamic conversions into additional stage-specific subtypes/states. Most dynamic changes occur at 3-days post-injury and by day-14 the second wave of microglial activation emerged, accompanied with changes in various cell types including neurons, indicative of the second round of attacks. By day-38, major cell types are still substantially deviated from uninjured states, demonstrating prolonged alterations. This study provides a comprehensive mapping of cellular/molecular pathological changes along the temporal axis after SCI, which may facilitate the development of novel therapeutic strategies, including those targeting microglia.

## Introduction

Traumatic injuries to the central nervous system (CNS) including the spinal cord often result in permanent loss of sensory, motor, and autonomic functions, without effective treatment. Unlike nerve injury of the peripheral nervous system (PNS), where severed axons may grow back into the distal nerve sheath after Wallerian degeneration, and reinnervate peripheral targets, adult CNS axons do not regenerate well. There are two major known hurdles: (1) attenuated axonal regeneration capacity of adult CNS neurons, and (2) inhibitory influences from CNS myelin debris as well as solid physical barriers in the form of astroglial scars. Although the spinal cord injury (SCI) research field has been focusing on promoting axonal regeneration for years, which is a similar thinking paradigm as for PNS neural repair, new types of CNS repair strategies have recently emerged. There have been multiple reports describing enhanced SCI repair, not just through long-distance axonal regeneration, but through building new local relay-neural circuits either by implementing exogenous neural stem/progenitor cells (NSCs) or engaging endogenous NSCs.^1–3^ Although this new repair strategy also created a microenvironment that would facilitate axonal outgrowth, the new strategy is much more complicated, involving new neurogenesis, subsequent neuronal maturation, synaptogenesis, neural circuit formation, and neural plasticity. These are biological processes distinct from axonal regeneration per se, and with additional hurdles to overcome.^1–5^

Current knowledge about the injury microenvironment after SCI is still rather limited.^6–10^ Quite dynamic cellular and molecular changes occur in a temporally-specific manner. Breakage and re-establishment of blood vessels and blood–brain barriers (BBB) involve dynamic gene expression changes in endothelial cells, pericytes, and astrocytes.^11,12^ Neural damages involve neural inflammation, synaptic dysfunction, neuronal degeneration, cell death, demyelination, reactivation of NSCs, astrogliosis, etc., which are complicated and influenced by temporal and spatial-specific factors.^13^ These events in concert participated in the formation of a dynamic injury microenvironment, impeding the neural regeneration process. Understanding these processes in detail may facilitate the development of different therapeutic approaches targeting different pathological processes with optimized timing.

The spinal cord injury research field has suffered a great deal from variable models and irreproducible results. It is important to standardize SCI modeling by standardizing surgical procedures and animal species. Moreover, more universal criteria, ideally based on “big data” processing, to objectively evaluate the validity and stability of the SCI models will greatly benefit the SCI research field, so that cross-platform/laboratory comparisons could be possible. Toward this, transcriptomic analyses of the spinal cord tissues, at different time points post-injury, had been postulated to objectively reveal the various molecular cellular pathological events caused by SCI.^8–10,14^ However, bulk-RNA sequencing only provides averaged gene expression from all, yet diverse cell types in spinal cord tissues, which could not precisely reveal specific changes from each cell type. Single-cell mRNA sequencing (scRNA-seq) exactly overcomes such a hurdle,^15^ and with proper tissue dissociation and single-cell suspension preparation methods, is capable of demonstrating the molecular cellular events with single-cell resolution, in normal control and injured spinal cord tissues at different times after injury.

In the present study, we carried out scRNA-seq analyses on both male and female adult C57BL/6 mice uninjured as well as injured spinal cord tissues at different time points post-injury. Data revealed most dynamic changes occurring in microglia, astrocytes, endothelial cells, and also pathological changes in neurons. In addition, both microglia and astrocytes appeared to have multiple subtypes in the uninjured spinal cord, which underwent dynamic changes post-injury. Microglia activation appeared to have two waves with the second wave occurring at 14 days post-injury, which coincided with secondary injuries to neurons. Interestingly, while changes in all other cell types tended to revert back to that of the uninjured state, microglial cells appeared to permanently shift to different states, leading to more long-term alterations of the spinal cord immune microenvironment. Interestingly, microglial cells that carried features of regeneration-promoting microglia in neonatal mice,^16^ were also detected in adult mouse spinal cord post injury, yet with subtle differences in gene expression as compared to their neonatal counterparts. For example, they expressed high levels of *Cd68*, which may inhibit the regeneration-promoting function of this microglial subtype.^16^ We hope that this study will serve as a prototype template reference, systematically describing the cellular and molecular pathological events in mouse spinal cord after crush injury, based on which we discussed their relevance to clinical observations after SCI, optimal timings for different therapeutic approaches. Our results also suggested that microglia might be the next line of major therapeutic targets to treat SCI.

## Results

### Spinal cord crush injury in male and female C57BL/6 mice

**Fig. 1:**
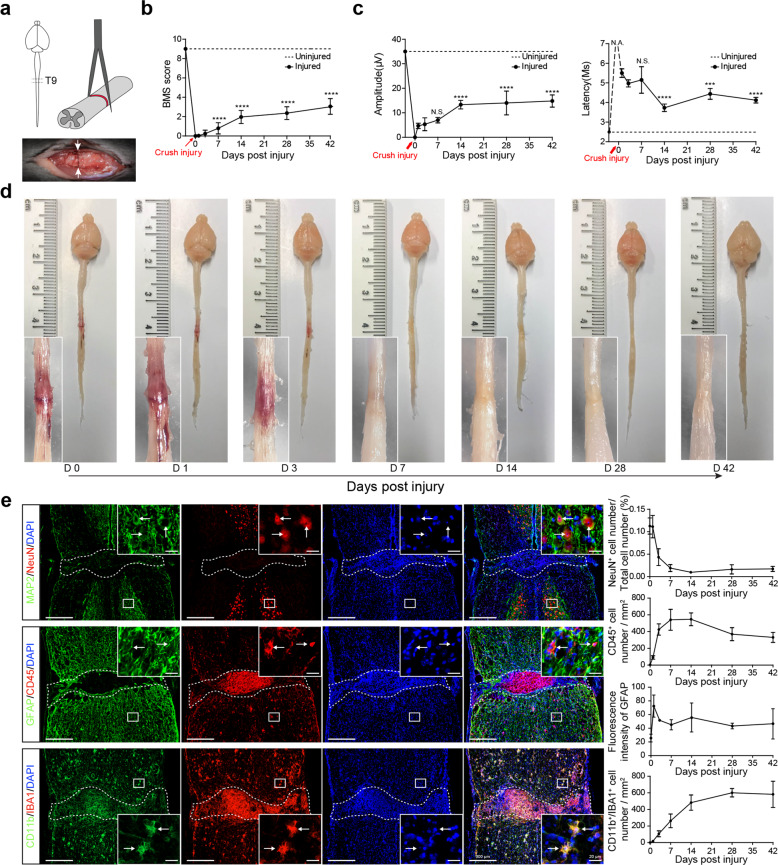
Classic characterizations of the C57BL/6 mouse spinal cord crush injury model. **a** Schematic illustration of the anatomical positioning of the crush injury site (upper left diagram) using a pair of forceps with crush time being 3 s (upper right diagram). The lower panel shows an example of the gross anatomy of spinal cord tissue immediately post-injury. **b** BMS open-field test to show hindlimb motor functional recovery from 0 to 42 d post-SCI (mean ± SEM; ****P < 0.0001, *n* ≥ 20, unpaired Student’s *t*-test with comparisons between each time point to 1 d post-injury). **c** Electrophysiological analysis within 6 weeks post operations, two panels show MEP amplitude and latency changes (mean ± SEM, *n* ≥ 3, ****P < 0.0001, ***P < 0.001, unpaired Student’s *t*-test with comparisons between each time point to 1 d post-injury; N.A., not applicable, N.S., not significant). **d** Gross anatomical analysis to show lesion core changes from 0 to 42 d. **e** Immunofluorescent staining of 42 d spinal cord tissue with neuronal markers MAP2/NeuN, an astroglial marker GFAP, and pan leukocyte and microglial markers CD45, IBA1, CD11b. The right panels demonstrate the quantitative analysis of fluorescent signals within 1 mm range encompassing the lesion core (mean ± SEM, *n* ≥ 3)

This study was designed to establish stable mouse crush injury-based SCI models to allow for systemic profiling of the molecular and cellular pathological changes as well as anatomical, electrophysiological, and behavioral alterations post SCI. The surgical site was at T9 of C57BL/6 male and female mouse spinal cord using forceps with tip size being 0.2 mm and crush time being 3 s^12,17^ (Fig. 1a). Such an injury consistently led to motor behavioral deficits as measured by Basso Mouse Scale (BMS) scoring in a double-blinded manner. By day 7 post-surgery, small but statistically significant increases in BMS scoring were observed (Fig. 1b). Motor function continuously increased until day 14. Interestingly after day 14, the recovery rate appeared to slow down, which was also reflected by motor-evoked potential (MEP) in both amplitude and latency (Fig. 1c). A total of more than 160 mice were monitored for behavioral changes and for each time point more than 20 mice were analyzed. Small error bars were indicative of small variations in our SCI model. Gross anatomical analysis and H&E staining indicated that edema was prominent soon after the operation and lasted until day 3 post injury (Fig. 1d, and Supplementary Fig. 1a). By day 7, edema started to resolve, but tissue degeneration or scarring at the lesion site was apparent, which lasted until day 42 (Fig. 1d, and Supplementary Fig. 1a). Immunofluorescent staining with neuronal markers MAP2/NeuN, an astroglial marker GFAP, as well as pan leukocyte and microglial markers CD45, IBA1, CD11b, indicated dramatic decreases in neuronal populations in the epicenter of the lesion core during the first week after injury, which was accompanied by rapid increases in numbers of CD45^+^ cells (Fig. 1e, and Supplementary Fig. 1b–d). CD11b and IBA1 double-positive microglia in the epicenter also increased following injury and plateaued after 28 days post-SCI (Fig. 1e, and Supplementary Fig. 1b–d). GFAP^+^ reactive astroglia rapidly increased post-SCI, peaked around day 1, followed by a decrease, yet remained elevated above noninjury levels until 42 days post-SCI (Fig. 1e).

### Population-based- and scRNA-seq revealed consistent temporal transcriptomic changes reflecting critical pathological events after SCI

**Fig. 2:**
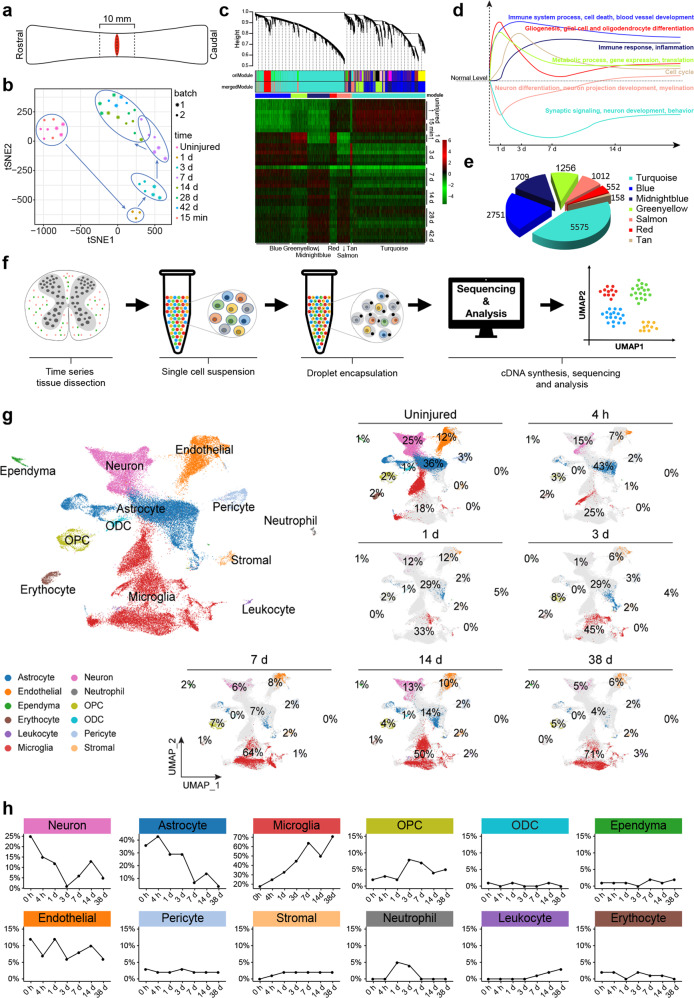
Temporal population-based (bulk-RNA-seq) and scRNA-seq uncovered transcriptomic changes post-SCI. **a** Schematic illustration of tissue sampling for bulk-RNA-seq and scRNA-seq. **b** The t-SNE plot visualizes 39 samples from 2 bathes, demonstrating consistent sequencing results from 2 batches. **c** Hierarchical clustering dendrogram of 39 samples shows coexpression modules identified using WGCNA. Seven modules were detected post 0.25 threshold merge. An expression heatmap of each module across all samples is demonstrated. **d** GO terms of 7 gene modules and their averaged gene expression temporal changes of post-SCI. **e** Pie-chart demonstrating gene counts of each module. **f** Summary of 10× Genomics scRNA-seq experimental workflow. **g** UMAP visualization plot of 59,558 spinal cord cells sequenced from all samples, color-coding defined 12 major cell types based on signature gene expression. The panels on the right show the proportion of each cell type at each time point before and post SCI. **h** Line charts demonstrating temporal changes of the relative contents of the 12 major cell types detected through scRNA-seq

Spinal cord segments, 10 mm in length, encompassing the lesion site were dissected and subjected to bulk RNA sequencing (Fig. 2a). Two-dimensional principal component analysis (PCA) of a total of 39 samples from two batches of experiments revealed clustering of uninjured and 15 min post-injury spinal cord samples, which were distinct from those at other times after injury (Fig. 2b). Moreover, a temporal signature appeared to be present, indicative of specific pathological events occurring at distinct times post-injury (Fig. 2b). Weighted Gene Coexpression Network Analysis (WGCNA) revealed 7 gene modules enriched for genes involved in different biological processes. Two gene modules (Turquoise and Salmon) associated with “synaptic signaling”, “neuron development”, “behavior”, “neuron differentiation”, etc., rapidly decreased expression after SCI and tended to recover after reaching the lowest point at day 7 and day 1, respectively (Fig. 2c–e). Two immune function-related modules (Blue and Midnight Blue) increased expression following SCI and remained at high levels of expression until 42-days post-injury. A “gliogenesis” related module (including astrocytic, oligodendrocytic, as well as microglial lineages) (Red) first went up after injury and dropped by day 3, yet remained above uninjured levels thereafter. A typical “cell cycle” module (Tan) increased post-SCI, peaked by day 3, and then gradually decreased. The last module (Greenyellow) representing “metabolic processes”, “gene expression”, “translation” also rapidly increased expression post-injury, peaked at day 1, and gradually decreased, yet remained above control levels thereafter (Fig. 2c–e).

Although WGCNA helped interpretation of bulk-RNA-seq data, so that temporal changes of major pathological events could be objectively reflected by transcriptomic analyses, many cell-type-specific changes were still masked through bulk-sequencing. To circumvent this issue, we performed high-throughput scRNA-seq using the 10× Genomics platform (Fig. 2f). Both male and female C57BL/6 mice were used in the analyses, 10 mm-long spinal cord segments encompassing the lesion site were dissociated into single cells through our proprietary method developed based on published studies.^18,19^ For non-injury controls, male and female spinal cord samples were sequenced separately, and male and female scRNA-seq data overlapped rather well (Supplementary Fig. 2a), indicating consistency of the scRNA-seq method and also providing a rationale for pooling male and female tissues together for scRNA-seq, particularly regarding samples at different time points after injury. A total of 59,558 cells were sequenced (Fig. 2g), 12 cell types were defined based on the expression of cell-type-specific marker genes (Supplementary Fig. 2a, b). Time point-specific scRNA-seq data were also presented (Fig. 2g), and changes of relative cell content of each of the cell types were illustrated in Fig. 2h. Among the 12 cell types, stromal cells, neutrophils, and lymphocytes were not present in the uninjured spinal cord but appeared in the spinal cord at specific times post-injury. Stromal cells entered the spinal cord at 4 h post-injury, plateaued by day 1, and remained at the same level until 38 days after injury. Neutrophils entered into the spinal cord and peaked at day 1–3 post-injury, then disappeared by day 7. CD3 positive lymphocytes appeared in the spinal cord only after day 3 post-injury and continued to increase in number until at least 38 days post-injury (Fig. 2h). Among the remaining 9 cell types, ependymal cells, oligodendrocytes (ODC), pericytes, and erythrocytes appeared to have smaller changes as compared to the rest of the 5 cell types. Oligodendrocyte progenitor cells (OPCs) changed more dramatically between 3–7 days post-injury, and endothelial cells appeared to have constantly undergone remodeling as indicated by increases and decreases in numbers (Fig. 2h). The most dynamically changing cell types were neurons, astrocytes, and microglia, with neurons and astrocytes, decreased, whereas microglia increased in relative cell contents. Interestingly, on day 14, microglia had a second wave of increase, which coincided with the second drop of neuronal and astrocytic cell contents (Fig. 2h). In support of this, we plotted expression of a major pro-inflammatory factor, TNFα,^20^ and found that after the first rise there appeared to be a second rise occurring after 2 weeks post-injury (Supplementary Fig. 2c), which was also reported by Chio et al.^21^

To investigate the relationship between scRNA-seq and bulk-RNA-seq data, we analyzed seven modules of genes from the aforementioned WGCNA. The averaged expression of the top 20 hub-genes of each module was highlighted in the scRNA-seq UMAP plot and their expression time courses in bulk- and scRNA-seq data sets were simultaneously plotted (Supplementary Fig. 2d). Strikingly, the “Midnight-blue” module with GO terms including “immune response”, “inflammation”, “microglia migration” was highly expressed in the microglia population based on scRNA-seq (Supplementary Fig. 2d), suggesting this WGCNA module was microglia-specific. Similarly, the “Turquoise” module representing synaptic signaling was highlighted in the neuronal population, indicating this module was neuronal-specific. The “Tan” module, which was “cell-cycle” specific, highlighted one subpopulation in astrocytes, and one in microglia, suggesting these two cell types were the major mitotic cell populations in the spinal cord after injury. In contrast, the “Green-yellow” module, representing general metabolic processes lit up almost all cell populations and did not show cell-type preferences, which was expected. Lastly, the “gliogenesis” and “nucleoside metabolic process”-related “Red” module also lit up many cell populations including astrocytes, OPC, and microglia. Overall, the two sets of data gave relatively similar results, indicating the validity of scRNA-seq data as well as the consistency of the SCI model.

### Dynamic changes in spinal cord neuronal populations after SCI

**Fig. 3:**
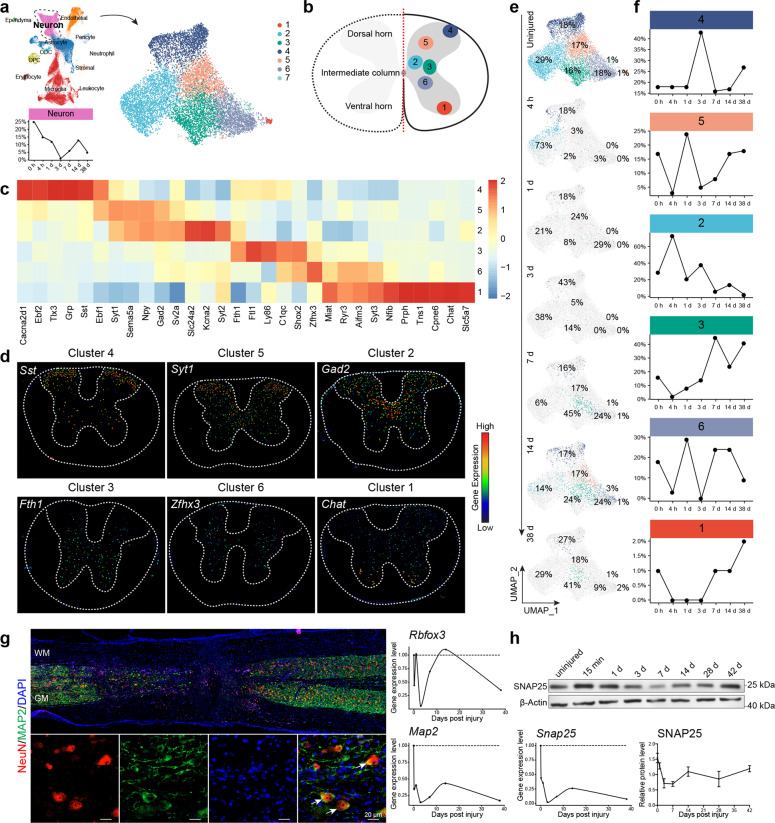
Dynamic changes in spinal cord neuronal populations post SCI. **a** UMAP visualization plot showing seven neuronal clusters (subtypes). **b** Schematic diagram of the spatial positioning of the six main neuronal clusters in the spinal cord based on signature gene expression. **c** Heatmap of normalized mean expression of signature genes for each neuronal cluster. **d** Color-coded in situ images of selected cluster-specific gene expression downloaded from the Allen Brain Atlas website. **e** UMAP plots indicating temporal changes of spinal cord neuronal clusters (subtypes). **f** Line charts demonstrating temporal changes of the relative contents of the six major spinal cord neuronal clusters. **g** Immunofluorescent staining of 3 d spinal cord tissue with neuronal markers MAP2/NeuN (*Rbfox3*). The panels on the right show the temporal changes of *Rbfox3* and *Map2* gene expression changes based on scRNA-seq. **h** Western bolt analyses of SNAP25 protein in the spinal cord at different times post SCI. The lower left panel demonstrates changes in SNAP25 mRNA, and the lower right panel shows changes in SNAP25 protein (mean ± SEM, *n* ≥ 3)

ScRNA-seq data revealed six major neuronal cell types in the uninjured spinal cord (Fig. 3a). Cluster 7 was omitted for further analyses because it might represent doublets as it expressed both neuronal and microglial genes (Supplementary Fig. 3a). Based on specific gene expression profiles, cluster 1 neurons were defined as cholinergic motor neurons located in the ventral horn because they expressed *Chat*, *Slc5a7*, *Cpne6*, as well as classic spinal motor neuron markers *Isl1* and *Mnx1* (HB9)^15,22–24^ (Fig. 3b–d, and Supplementary Fig. 4a, b), whereas cluster 4 and 5 carry typical glutamatergic spinal sensory neuron features, with an expression of *Tlx3*, *Grp*, *Sst*, *Ebf2* for cluster 4 and *Ebf1*, *Sema5a*, *Npy*, *Syt1* for cluster 5^15,22–24^ (Fig. 3b–d). Based on prior knowledge,^15,22–24^ cluster 4 neurons should be located in the dorsal most area, and cluster 5 neurons should be just below cluster 4 (Fig. 3b–d). Cluster 2 neurons expressed a number of featured genes like cluster 5 including *Npy* and *Gad2*, suggesting these two clusters of neurons were closely related. On the other hand, cluster 2 neurons also specifically expressed *Kcna2*, *Slc24a2*, and *Syt2*, suggesting that they were GABAergic interneurons located in the intermediate zone along the dorsal-ventral axis^22,25^ (Fig. 3b–d). Cluster 3 expressed *Fth1*, *Ftl1*, *Shox2*, and cluster 6 expressed *Shox2*, *Zfhx3*, *Nfib*, and *Syt3*. Based on the literature, spinal cord ventral interneurons could be divided into V1 (En1^+^), V2 (Lhx3^+^), and V3 (Sim1^+^) interneurons, amongst which the V2 population could be further divided into V2a (Vsx2/Chx10^+^ and Sox14^+^), V2b (Gata3^+^), V2c (Sox1^+^) subtypes. We plotted the expression of these marker genes, and data indicated that clusters 2, 3, and 6 appeared to contain ventral interneuron features.^22–24^ However, each cluster of clusters 2, 3, or 6 might still be a mixture of previously defined ventral interneuronal subtypes (Fig. 3b–d, and Supplementary Fig. 4c, d). Interestingly, the dorsal–ventral positioning of these 6 clusters of neurons appeared to be reflected by their featured gene expression, such that neurons positioned next to each other shared expression of more featured genes or with more similar transcriptomes (Fig. 3c, d, and Supplementary Fig. 3b).

Before the injury, all six clusters of neurons could already be detected. Four hours after SCI, the relative numbers of ventral neurons (clusters, 3, 6, 1) as well as cluster 5 neurons already decreased dramatically, while cluster 2 interneurons and cluster 4 sensory neurons appeared to be more resistant to SCI at this early time point (Fig. 3e, f). On day 3 post-injury, when the total neuronal population almost dropped to zero (Fig. 2h, and Fig. 3a), clusters 2–4 appeared to be still detectable in very small quantities. By day 14, miraculously, all 6 clusters of neurons appeared to all return with relative ratios similar to the uninjured state, yet, after day 14, the second drop of the neuronal population was apparent, with clusters 2 and 6 having dropped most dramatically, while 4/3 appeared to be more resilient (Fig. 3e, f).

This finding was rather unexpected because, at day 3 post-SCI, scRNA-seq results indicated almost no neurons could be detected, whereas by immunohistochemical analyses, there were still plenty of NeuN (*Rbfox3*) and MAP2 double-positive neurons detected in the 1 cm long specimen, which was used for scRNA-seq (Fig. 3g). It was possible that spinal neurons, while still containing NeuN/MAP2 proteins, had already substantially shut down their global gene expression, such that they became undetectable by scRNA-seq. The current scRNA-seq technology only has an mRNA capture rate of less than 50%, leading to the limitation in the detection of moderately expressed genes. Consistently, another neuronal marker SNAP25 mRNA dropped to almost zero by scRNA-seq, whereas the SNAP25 protein remained close to 50% of non-injury levels (Fig. 3h). The discordant regulation between neuronal gene mRNA and proteins might explain why by day 14 almost all neurons became detectable again after everything appeared to be all lost by day 3. This was likely not due to regeneration but the “awakening” of neurons. We proposed that after SCI, spinal cord neurons underwent a “shock response”, during which they shut down most of their gene expression, and therefore were no longer detectable by scRNA-seq. Such a “shock” peaked around day 3. Subsequently, neurons were gradually revived (awakened) and returned back to their normal gene expression state by day 14. This event might correspond to the well-known clinical term “spinal concussion/shock”. It is worth noting that after day 3 when neurons started to revive, animal walking behavior, as well as MEP, also started to recover (Fig. 1b, c).

### Diverse astrocytic subpopulations converged after SCI

**Fig. 4:**
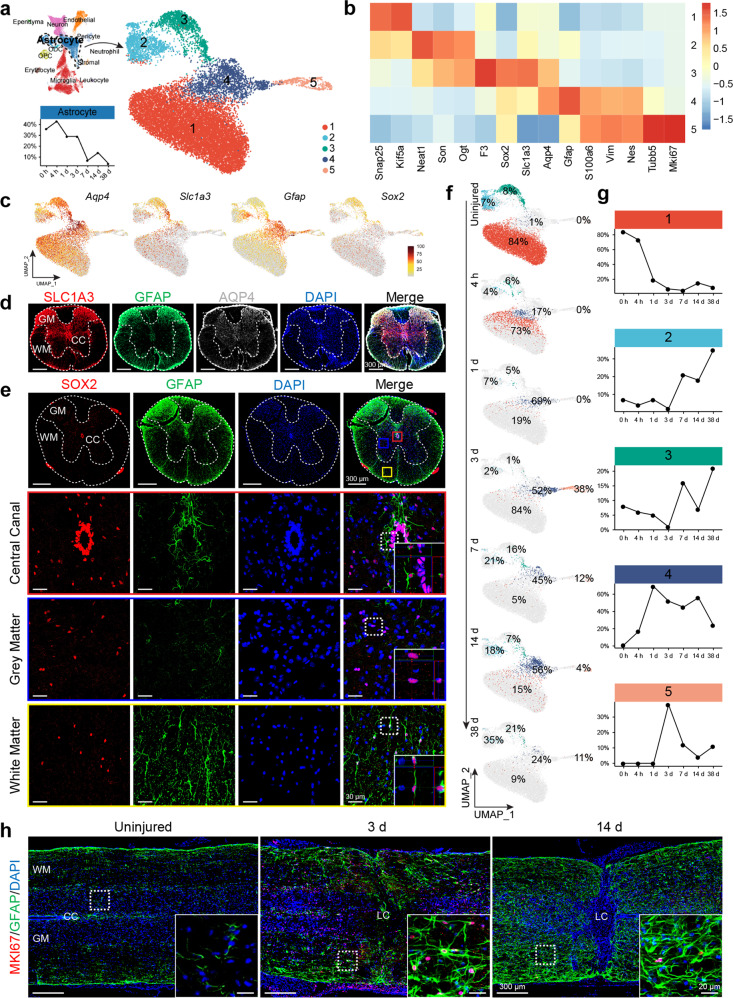
Diverse astrocytic subpopulations converged post-SCI. **a** UMAP visualization plot showing 5 astrocyte clusters (subtypes). **b** Heatmap of normalized mean expression of signature genes for each astrocytic cluster. **c** Feature plots showing gene expression of *Aqp4*, *Slc1a3*, *Gfap*, and *Sox2* in different astrocyte clusters. **d** Immunofluorescent staining of uninjured spinal cord tissue with antibodies against SLC1A3, GFAP, and AQP4. **e** Immunofluorescent staining of uninjured spinal cord tissue with GFAP and SOX2, showing that SOX2 positive cells were concentrated surrounding the central canal, and also sporadically in gray and white matters; in the white matter, all SOX2 positive cells were also positive for GFAP, whereas in the gray matter only some SOX2 positive cell were GFAP positive. SOX2 positive cells lining the central canal were GFAP negative. **f** UMAP plots indicating temporal changes of spinal cord astrocyte clusters (subtypes). **g** Line charts demonstrating temporal changes of the relative contents of the five major spinal cord astrocyte clusters. **h** Immunofluorescent staining of spinal cord tissue 3 days post-injury with Gfap and cell proliferation marker Mki67

The astrocytic population could be divided into 5 clusters, three of which (clusters 1–3), already existed in the uninjured spinal cord. Cluster 1 was a quite large subpopulation, and surprisingly also expressed a number of neuronal-trait-associated marker genes including *Snap25* and *Kif5a*. Of course, this did not mean that these gene-encoded proteins were necessarily being expressed. Cluster 2 astrocytes were related to cluster 1 in that they also expressed *Snap25* and *Kif5a*, though at lower levels than in neuronal populations. In addition, cluster 2 expressed *Neat1*, *Son*, and *Ogt*, which were also expressed in cluster 3 astrocytes (Fig. 4a, b, f, g). Cluster 3 astrocytes, surprisingly expressed a neural stem/progenitor cell marker, *Sox2*. Cluster 4/5 astrocytes were only detected in the spinal cord after SCI, and they were *Vim* and *Gfap* expressing, with low levels of *Sox2* expression. Cluster 5 astrocytes were dividing as they were *Mki67* positive and were distributed in a gradient, such that more dividing cells were located closer to the injury epicenter (Fig. 4a–c, f, g, and Supplementary Fig. 5a).

To determine the spatial distribution of the three astrocytic subtypes in the normal spinal cord, we performed immunohistochemical analyses. We found that AQP4 were pan astrocytic and AQP4^+^ astrocytes were relatively evenly distributed in both white and gray matters. GFAP, on the other hand, was highly expressed in white matter, surrounding the central canal, as well as dorsal lemniscus (Fig. 4a–d). A relatively astrocyte-specific glutamate transporter *Slc1a3* (EAAT1/GLAST), which was sporadically expressed in all three clusters of astrocytes, also appeared to be primarily expressed in the dorsal gray matter, surrounding the central canal, and with some in the white matter (Fig. 4a–d). Immuno-co-labeling of SOX2 and GFAP indicated that SOX2 positive cells were concentrated surrounding the central canal, and also sporadically in gray and white matters. Interestingly, in the white matter, all SOX2 positive cells were also positive for GFAP, whereas in the gray matter only some SOX2 positive cells were GFAP positive, and SOX2 positive cells lining the central canal were GFAP negative (Fig. 4e). Given that cluster 1 astrocytes expressed many neuronal genes, we assumed that they might be primarily gray matter astrocytes, whereas white matter astrocytes were likely enriched in clusters 3 or 2, even though clusters 3 and 2 astrocytes could also appear in the gray matter.

After SCI, different astrocyte subpopulations appeared to all become reactive astrocytes, which were positive for *Vim*, *Gfap* (cluster 4/5). Cluster 4/5 cells appeared to be one type of cells with some in the mitotic phase (cluster 5) and some not (cluster 4). Since Ben Barres’s group reported that reactive astrocytes could be divided into *C3* expressing type A1 cells and *S100a10* expressing type A2 cells,^26^ we examined the expression of *C3* and *S100a10* in cluster4/5. We discovered that cluster 5 dividing astrocytes expressed *S100a10* but not *C3*, whereas cluster 4 astrocytes could be potentially slipt into type A1 (*C3*) and A2 subtypes, both of which were only detected after SCI (Supplementary Fig. 5f). By day 3 post-SCI, clusters 1–3 astrocytes reached the lowest levels in the 1 cm segment of the spinal cord encompassing the lesion site, either dead and/or converted into cluster 4/5 astrocytes (reactive astrocytes). Mitotic astrocytes peaked also at day 3 post-SCI (Fig. 4f–h, and Supplementary Fig. 5a–c). Reactive astrocytes showed stellate morphology at day 3 post-injury but were excluded from the lesion core. However, by day 14 heightened GFAP immunofluorescent signals were detected in the spinal cord across both white and gray matters, with astrocytes containing thick processes and becoming interconnected forming astrocytic nets.

Whole transcriptome clustering results again indicated that cluster 5 mitotic astrocytes were least similar to the rest of the clusters (1, 2, 3, 4), amongst which cluster 4 was most similar to cluster 5. On the other hand, cluster 4 astrocytes were most similar to the rest of the astrocytes as a whole, suggesting convergence of clusters 1–3 to cluster 4 was possible. In addition, clusters 1 and 4 were the most alike, and clusters 2 and 3 were more similar (Supplementary Fig. 3c).

### Dynamic alterations of the microglia populations after SCI

**Fig. 5:**
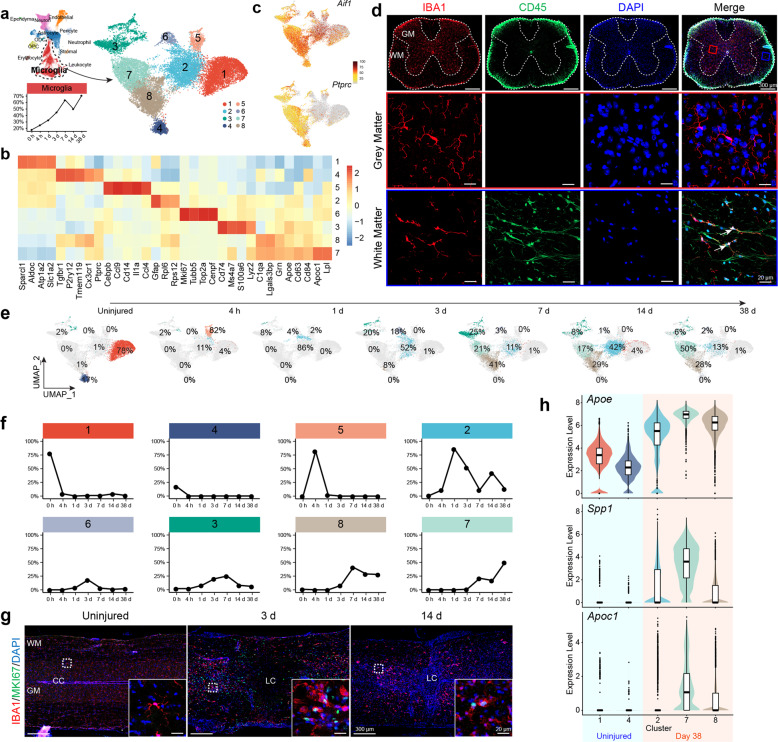
The microglia population diversified post-SCI. **a** UMAP plot showing eight microglia clusters (subtypes). **b** Heatmap of normalized mean expression of signature genes for each microglial cluster. **c** Feature plots showing gene expression of *Aif1* (IBA1) and *Ptprc* (CD45) in different microglia clusters. **d** Immunofluorescent staining of uninjured spinal cord tissue with IBA1 and CD45, showing that IBA1/CD45 double-positive cells only settled in the white mater, but IBA1 positive cells in the gray matter were CD45 negative. **e** UMAP plots indicating temporal changes of spinal cord microglia clusters (subtypes). **f** Line charts demonstrating temporal changes of the relative contents of the eight major spinal cord microglia clusters. **g** Immunofluorescent staining of spinal cord tissue 3 days post-SCI with microglia marker IBA1 and cell proliferation marker MKI67. **h** Violin plots showing expression of Alzheimer’s Disease-related microglial signature genes *Apoe*, *Spp1*, and *Apoc1* in microglia cluster 1/4 (uninjured) and cluster 2/7/8 (38 d post-SCI)

**Fig. 6:**
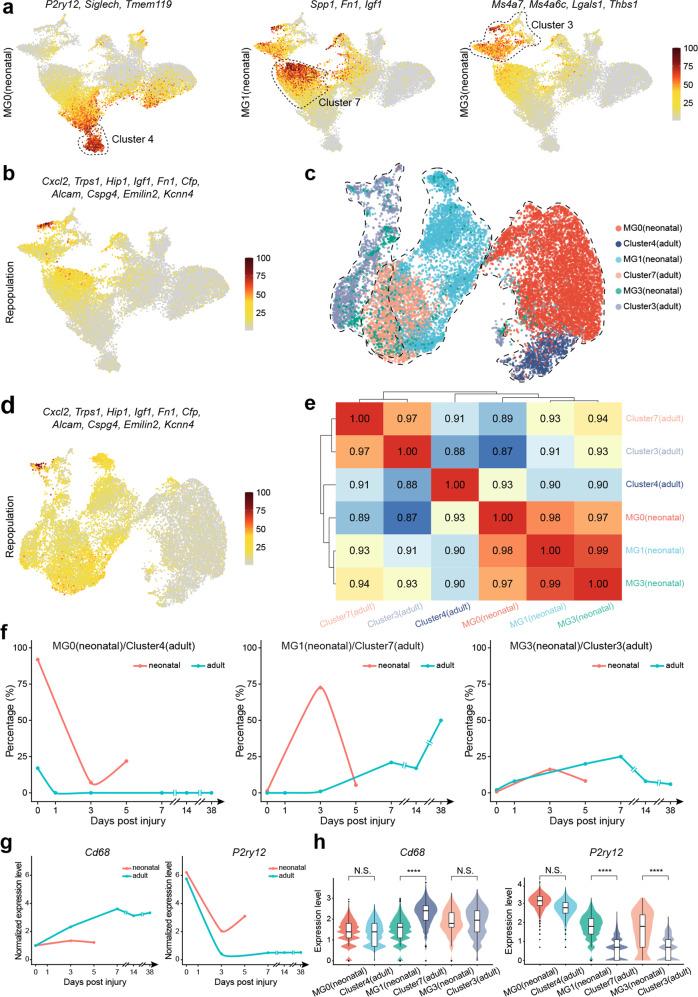
Presence of regeneration-promoting microglia in the adult spinal cord. **a** Feature plot showing average expression of different groups (MG 0/1/3) of neonatal microglia signature genes in different adult spinal cord microglia clusters. **b** Feature plots showing average expression of repopulation microglia signature genes in adult spinal cord microglia clusters. **c** UMAP visualization plot showing neonatal microglia MG 0/1/3 and adult spinal cord microglia cluster 4/7/3 merged correspondingly, in a subtype-specific manner. **d** Feature plots showing average expression of repopulation microglia signature genes in MG 0/1/3 (neonatal) and cluster 3/4/7 (adult). **e** Clustering heatmap based on Pearson’s correlation coefficient between MG 0/1/3 (neonatal) and cluster 3/4/7(adult), showing similarity between corresponding neonatal and adult microglia subtypes were 0.93, across all three subtypes. **f** Line chart showing temporal changes in relative contents of MG 0 (neonatal)/cluster 4 (adult), MG 1 (neonatal)/cluster 7 (adult), and MG 3 (neonatal)/cluster 3 (adult). **g** Temporal gene expression changes of *Cd68* and *P2ry12* in neonatal and adult spinal cord post-injury. **h** Violin plots showing *Cd68* and *P2ry12* expression levels in MG 0 (neonatal)/cluster 4 (adult), MG 1 (neonatal)/cluster 7 (adult), and MG 3 (neonatal)/cluster 3 (adult) (****P < 0.0001; N.S., not significant)

The microglial population was the most abundant cell type detected in the spinal cord after SCI by our scRNA-seq (Fig. 5a). A total of 8 clusters of microglial subpopulations were detected (Fig. 5a, b). In the uninjured spinal cord, clusters 1 and 4 were the two major relatively homogenous microglial subtypes detected (Fig. 5a, e). A small proportion of the quite diverse cluster 3 microglia were also present in the normal spinal cord, which was *Mrc1* (CD206) and *Lyve1* expressing (Supplementary Fig. 6a). Some literature has suggested that *Mrc1* and *Lyve1* positive cells were peripheral macrophages that infiltrated into the spinal cord,^27^ and others have suggested that they were M2 (anti-inflammatory) type microglia as they also express *Cd163*^28,29^ (Supplementary Fig. 6b). Overall, this only represented a very small population (Fig. 6a, e, and Supplementary Fig. 5a, b). As one of the two major microglial populations detected in the uninjured spinal cord, cluster 4 expressed *Tmem119* and *Sall1* (Supplementary Fig. 6c), which suggested these subpopulations were classic microglia in the CNS and precluded them from being infiltrating or residential macrophages.^27,30,31^ Cluster 4 microglia were also positive for *Ptprc* (CD45), *Cx3cr1*, and *Aif1* (IBA1) (Fig. 5b, c). Immunofluorescent staining indicated that cluster 4 microglia were primarily located in the white matter (Fig. 5d). Interestingly cluster 1 microglial represented a larger microglial population (78%) (Fig. 5e, f). It expressed *Aif1* (IBA1) but had very low (if any) expression of *Ptprc* (CD45) or *Cx3cr1* (Fig. 5b, c). They most likely represented gray matter microglia, because immunohistochemical analyses indicated that IBA1 positive cells in the gray matter were CD45 negative (Fig. 5d). In addition, cluster 1 microglia also expressed *Slc1a2* (GLT1/EAAT2), *Aldoc*, and *Sparcl1*, which were previously known to be astrocyte-specific markers^32^ (Fig. 5b).

Four hours after SCI, almost all microglia appeared to be converted into the cluster 5 subtype with high expression of chemokine genes including *Ccl4/9*, *Cd14*, *Il1a*, and *Cebpb* (Fig. 5b, e). Cebpb was a well-known master regulator for neural inflammatory responses.^33–35^ One day after the injury, cluster 5 subtype cells might be converted into cluster 2 subtypes, from which a mitotic subpopulation became cluster 6 cells (Fig. 5b, e). On day 1, cluster 3 cells also appeared to increase in relative numbers, becoming the second most abundant microglia population in the spinal cord (Fig. 5e, f), yet cluster 3 microglia at day 1 appeared to be pro-inflammatory as they reduced expression of the M2 type of microglial genes and increased expression of M1 genes (Supplementary Fig. 6d). On day 3, when the microglial cluster 2 was still the major microglial subtype, cluster 6 mitotic microglia peaked and cluster 5 disappeared (Fig. 5e–g). Similar to astrocytes, mitotic microglia were also present in a gradient, with larger numbers of dividing cells located closer to the epicenter (Supplementary Fig. 5a-e). Interestingly, while astrocytes were excluded from the lesion core, mitotic microglia were present within the lesion core (Supplementary Fig. 5c–e). In addition, cluster 3 further increased in number, and cluster 8 started to emerge (Fig. 5e, f). By day 7, the whole microglial population peaked, and major subtypes became clusters 7/8 and 2/3 (Fig. 5e, f). By day 14, there was a drop in the total relative microglial population in the spinal cord, which coincided with an increase in cluster 2 and decreases in clusters 3, 7, and 8 (Fig. 5e, f). By day 38, the total microglial population relative to total cells in the spinal cord increased again, with clusters 7, 8, and 2 being the major microglial subtypes (Fig. 5e, f). Based on the whole transcriptome, cluster 2 was overall more correlated with all other microglial subtypes (Supplementary Fig. 3d), making it possible that it was a key state converted from cluster 5 and gave rise to clusters 7/8 (Fig. 5e). Surprisingly, even by day 38 post-SCI, microglial populations were still deviated from those observed in the uninjured spinal cord (Fig. 5e, f), suggesting long-lasting alterations in the immune microenvironment after SCI. Consistent with this notion, we plotted expression of microglial genes associated with Alzheimer’s disease (AD),^36^ *Apoe*, *Spp1*, and *Apoc1*, in microglial cells before injury (Clusters 1, and 4) and 38 days after injury (clusters 2, 7, 8), and results indicated that after SCI microglial cells expressed higher levels of AD-associated genes and thus appeared to be in a more adverse/diseased state (Fig. 5h). Among the 8 detected microglial populations, cluster 3 appeared to be most heterogeneous and also changed dynamically at different times after SCI (Fig. 5e). It was possible that this cluster was a non-homogenous population, potentially containing infiltrating monocytes and/or macrophages from the periphery as well.

### Regeneration-promoting microglia in adult mouse spinal cord

In very recently published studies, microglia isolated from neonatal mouse spinal cord had been shown to promote spinal cord regeneration after SCI both in neonatal and adult spinal cords after transplantation.^16^ We found regeneration-promoting microglial genes such as *Fn1*, *Ms4a7*, *Thbs1*, and *P2ry12* were actually also expressed in adult spinal cord microglia before and/or after SCI (Fig. 6a). Moreover, it has been proposed that after microglia clearance, repopulated microglia in adult mouse CNS are also capable of promoting CNS regeneration after injury.^37^ We examined the expression of signature genes of repopulated microglia in adult mouse spinal cord after SCI and found that they were also highly expressed in clusters 3 and 7 (Fig. 5e, and Fig. 6b), as well as in neonatal mouse spinal cord microglia after SCI, where regeneration was prominent (Fig. 6c, d, and Supplementary Fig. 6f).

Parallel comparisons between our data and published neonatal spinal cord microglia data revealed that the majority (92%) of the resting microglia in the neonatal spinal cord was more similar to cluster 4 microglia in our data, with transcriptomic correlation coefficient being 0.93, which expressed both *Cx3cr1* and *Ptprc* (CD45) (Fig. 5b, Fig. 6a, c, e, and Supplementary Fig. 6g). Yet, they only contributed to 17% of uninjured adult spinal cord microglia (Fig. 5e, and Fig. 6f). The regeneration-promoting “bridging microglia” (MG3) in the neonatal spinal cord microglia study^16^ appeared to also exist in the adult spinal cord after injury, which expressed *Ms4a7*, *Ms4a6c*, *Lgals3*, and *Thbs1*, and were enriched in cluster 3 (Fig. 6a, c, and Supplementary Fig. 6g). The other regeneration-promoting microglia in the neonatal study (MG1) appeared to correspond to cluster 7 microglia in our study, which expressed *Fn1*, *Spp1*, *Igf1*, and *Clec7a* (Fig. 6a, c, and Supplementary Fig. 6g). However, cluster 7 microglia only emerged in the adult spinal cord 7 days after SCI, whereas in the neonatal spinal cord MG1 population peaked at day 3 (Fig. 6f).

Lastly, high levels of CD68 expression in microglia were negative, whereas high levels of P2ry12 expression were positive, correlated with spinal cord regeneration after injury.^16,38^ We, therefore, compared the expression of these two genes in microglia from our data (clusters 4, 7, and 3) and data from the neonatal study (MG 0, 1, and 3). It was apparent that adult microglia before injury had comparable levels of expression of these two genes as neonatal spinal cord microglia, whereas after spinal cord injury, adult microglia increased expression of *Cd68* and decreased expression of *P2ry12* (Fig. 6g, h), indicative of reduced regeneration-promoting power.

Taken together, it appeared that regeneration-promoting microglia, although still present in the adult spinal cord, were less abundant, appeared a bit later, and expressed higher levels of *Cd68* and lower levels of *P2ry12*, which likely dampened their ability to promote regeneration.

## Discussion

This comprehensive analysis was based on the establishment of a stable mouse spinal cord crush injury model, as indicated by behavioral indices of hundreds of animals and at different times after injury, as well as consistent transcriptomic data from both bulk-seq and scRNA-seq. Bulk sequencing of mouse spinal cord tissues after spinal cord crush injury overall revealed major pathological changes reflected by expression changes of gene modules, which included reduced expression of synaptic transmission-related genes, increased cell death, reactivation of cell cycle, likely from astrocytic and microglial lineages as judged by scRNA-seq, and activation of gliogenesis. In addition, increased expression of neural inflammation-related genes and injury/wound repair programs were also clearly indicated from bulk seq. Interestingly, these data were consistent with already published studies using mouse and rat SCI models, as well as other studies on the temporal cellular and molecular changes after SCI in rodents, indicative of conservation of molecular and cellular pathophysiology after SCI.^9,10,21,39^

ScRNA-seq revealed single-cell resolution data regarding the cellular and molecular responses after SCI, which was enlightening. It is well known that soon after SCI, patients enter a stage called “spinal shock” during which, there are no reflective responses below the level of injury.^40,41^ Clinically, this “shock” period could last from days, weeks, to even one month. After the “shock” period, neuronal activities could be restored and if triggered properly, may even initiate walking activities as in the case of “epidural stimulation”.^42,43^ The molecular and cellular bases for “spinal shock” were not fully understood. It is only known that neurons stop firing or neural transmission soon after injury. Our scRNA-seq study might open a channel for future detailed investigations regarding the cellular and molecular mechanisms underlying the “spinal shock” event because our data allow us to first postulate that “transient shutting down of global transcription in neurons” might be a hallmark for “spinal shock”. The pathological meaning of such a “shock” and whether or not prevention of such a “shock” would be beneficial to neural repair and functional recovery remained to be determined.

Acute excitotoxicity after SCI is known to occur very early, within 15 min to 3 h after SCI, during which glutamate levels are known to increase transiently to excitotoxic levels in the extracellular space.^44^ Since our data had the 4-hour time point, we specifically examined the expression of the neurotransmission-related program (the top 32 turquoise module hub genes) as well as a well-known key calcium responsive immediate early gene, c-Fos, which is known to be upregulated due to glutamate excitotoxicity. Data indicated that there were increases in c-Fos and neurotransmission-related genes at 4 h after SCI, which then decreased to the lowest levels by day 3, and went up again afterward (Supplementary Fig. 7), indicating the end of “neuronal shock” occurred after day 3, which also fit well with the time scale of the rise of BMS scores.

Subtypes of astrocytes have been identified in the white matter (protoplasmic) and the gray matter (fibrous) in previous research, but there was no systematic description of their molecular markers.^31,45^ Our spinal cord scRNA-seq revealed at least three astrocytic subpopulations existing in the normal uninjured spinal cord. GFAP and SOX2 double-positive astrocytes seemed to be enriched in white matter and juxtapose to, but not lining, the central canal. Interestingly, all Sox2 positive cells in the white matter were also GFAP positive, yet in gray matter, GFAP positive cells, which may or may not be SOX2 positive, were enriched in the dorsal sensory region, where SLC1A3 (EAAT1/GLAST) was also heavily positive. SOX2 single positive cells were enriched in the lining of the central canal, which might have ependymal cell features as well as neural stem cell activities.^46^ Interestingly, the majority of astrocytic cells in the gray matter also express a number of neuronal genes, such as *Snap25*, *Meg3*, *Nefl*, *Nefm*, *Nefh*, *Kif5a*, etc., which might reflect their close relationship with neurons and synapses. After the injury, diverse astrocytic subpopulations appeared to converge into a state, characteristic of reactive astrocytes, which were vimentin, GFAP, and Nestin positive and were likely dividing. When cells were in the cell cycle (S, G2M), they belonged to cluster 5 and when they were in G0 or G1, they belonged to cluster 4 (Fig. 4a, b, h).

It is still unclear about the unique functions of SOX2 and GFAP double-positive astrocytes. Published reports suggested that these cells might act as neural stem cells (NSCs) to initiate cell division and differentiation.^47^ In addition, SOX2 positive ependymal cells, which might be also FOXJ1 and/or CD133 (Prominin1) positive, are another candidate NSC population in the spinal cord participating in SCI injury responses.^48,49^ Of course, SOX2 negative CD133 (Prominin1) positive cells sharing transcriptomic signatures of endothelial cells (*Flt1*, *Tek*, *Sox17*, *Cldn5*, and *CD34*) and pericytes (*Pdgfrb*, *Vtn*, and *Cspg4*) (Supplementary Fig. 3b, and Supplementary Fig. 8a), which also existed in the 4^th^ ventricle, were detected in our scRNA-seq data^46^ (Supplementary Fig. 8a–c). These cells might also be adult spinal cord NSCs.^46^ Lastly, whether microglial cells, the other major proliferating cell types like astrocytes,^50^ also have some potential to function as NSCs remained to be determined. At least upon forced NeuroD1 expression microglia could be converted into neurons.^50^

In the epicenter, we found major cell types being CD45 positive infiltrating white blood cells or residential microglia as well as infiltrating fibronectin and collagen 1a2 positive stromal/fibroblastic cells (see Supplementary Fig. 9). Michael Sofroniew from UCLA reported that the astroglial (GFAP^+^) scar is involved in limiting inflammatory cells (CD45^+^) in the epi-center rather than spreading into the non-primary lesion areas. GFAP specific inhibition of the JAK-STAT pathway impaired glial scar formation causing inflammatory cells to spread and resulting in worse behavioral outcome.^51^ It seems that reactive astroglia and CD45^+^ inflammatory cells might be mutually repelling. In addition, stromal/fibroblastic cells might also repel astrocytes to enter the epi-center. The future full body of detailed mechanistic studies will be needed to answer this series of questions. It was also quite striking that within the 10-mm-long spinal cord segment, not just close to the epicenter of the injury, all astrocytes converted into reactive astrocytes, suggesting that the injury signal spread quite a long distance. Given that essentially every astrocyte has at least one endfeet associated with blood vessels, it is reasonable to assume that the injury signal(s) might come from the vasculature or blood supply. Of course, the other caveat is that the current 10× Genomics-based high throughput scRNA-seq technology still has some limitations in that less than 50% of mRNAs expressed in a cell could be captured and detected in scRNA-seq. Therefore, highly expressed gene sets might mask differences of moderately expressed yet important genes, and induction of strong injury responsive genes might allow cells to be grouped as seemly “similar” cells, rather than perhaps in reality, “different” cells.

Microglia were the most dynamically changing cell population in the spinal cord after SCI. Even by day 42 when injury responses tended to ease down and tissue homeostasis stabilized, the microglial populations still deviated from those before the injury, indicative of long-lasting alterations in the immune microenvironment. For example, Alzheimer’s Disease-related microglial signature genes were more prominently expressed in microglial cells isolated at day 38 after SCI^36^ (Fig. 5h), suggesting SCI resulted in a long-lasting adverse immune microenvironment. Transcription factor (TF) enrichment analyses of the bulk-sequencing data objectively revealed the most significant TFs (potentially master control genes) and their target genes acting at each specific time after SCI (Supplementary Fig. 10a, b). To our surprise, all these TFs are predominantly expressed in microglial cells, indicative of the critical roles of microglial cells in SCI responses. Indeed, in recently published studies, neonatal mouse microglial cells as well as “repopulating” microglia were shown to be strongly “regeneration-promoting”. Interestingly, transcription features represented by those “regeneration-promoting” microglial cells appeared to present in adult mouse spinal cord after injury with transcriptomic similarity reaching 93% (Fig. 6e). However, subtle, yet seemingly critical changes in gene expression, e.g., relatively higher-level expression of *Cd68* and lower-level expression of *P2ry12*, might limit the regeneration-promoting power of adult microglial cells. On the other hand, given the transplantation of neonatal microglial cells in the adult mouse, spinal cord after SCI could also promote regeneration of the adult spinal cord, which meant inhibitory influences of the adult spinal cord tissue microenvironment were insufficient to completely neutralize the function of “regeneration-promoting” microglia. Moreover, it has been reported that lowering the expression of CD68 in adult microglia could also promote regeneration.^16^ All these and our data, together, point out microglia being a new potential therapeutic target for CNS repair.

**Fig. 7:**
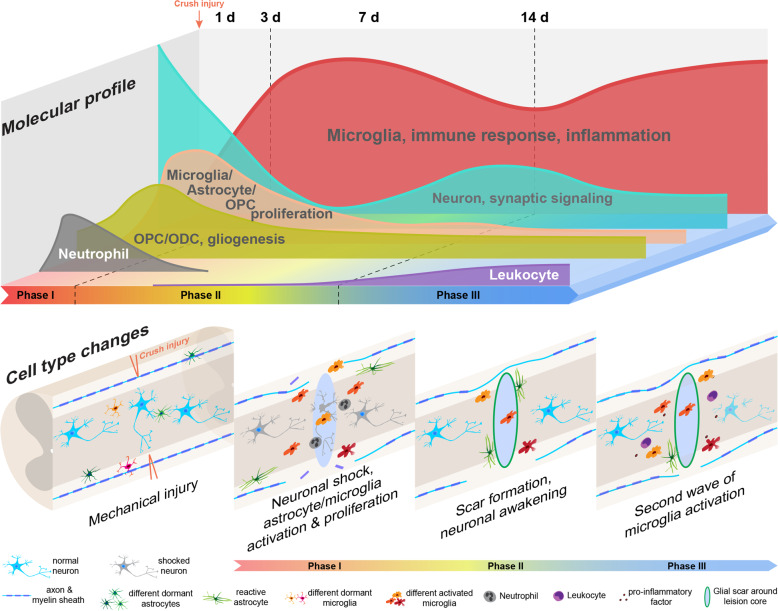
A schematic illustration of molecular and cellular changes post SCI

Lastly, both bulk-seq and scRNA-seq data demonstrated three critical periods after SCI, which corresponded well with clinical stagings after SCI^41,52^ (Supplementary Table 1, and Supplementary Fig. 11): 1) first 3 days after injury, rapid loss of neuronal functions, mitotic activation of astrocytes, oligodendrocyte progenitors, and microglia occurred, which were accompanied with dynamic changes of the vasculature endothelial cells, infiltration of neutrophil and stromal cells; 2) between 3 days and 14 days after SCI, a trend of recoveries took place for many cell types including neurons, which was accompanied with astroglial scar formation, infiltration of leukocytes, and further changes in vasculature endothelial cells; 3) after 14 days, reactivation of microglia and further decreases in neuronal, astrocytic, and endothelial populations, together with continued increases in leukocyte population were apparent (Fig. 7). This pathophysiological staging suggested that earlier (before day 3 in mouse) anti-inflammatory interventions might be key to prevent downstream chain reactions leading to adverse outcomes. In addition, prevention of early-phase “neuronal shock” might also be beneficial for reserving functional neuronal pools. The second wave of microglial activation two weeks after injury should also be attended, because this might play a critical role in subsequent and further spread and long-term continued injury to the spinal cord, further dampening the potential for regeneration. Whether this second wave of microglial activation is caused by infiltrating leukocytes remains to be determined. Through this study, we postulated that microglial cells should be a prominent target for the next phase of therapeutic development to treat SCI.

## Materials and methods

### Animal Experiments and ethics statement

All experimental procedures were approved by and performed in accordance with the standards of the Animal Welfare Committees of Tongji University in Shanghai, China, and the University of California, Los Angeles (UCLA), USA. Eight-week-old male and female C57BL/6 mice weighing between 18 and 20 g were used. All animal operations were performed under general anesthesia with inhalant isoflurane (2%) delivered in an oxygen-enriched air using a dissecting microscope (Nikon/Zeiss), and rodent stereotaxic apparatus (David Kopf). Laminectomy was performed at T9 to expose the spinal cord. No. 5 Dumont forceps (Fine Science Tools) fixed on stereotaxic apparatus were used to crush the spinal cord with persisting for 3 s. For in sham group, only laminectomy was performed on the T9 lamina without subsequent crush injury. Animals were monitored daily to avoid infection, aberrant wound healing, or weight loss. The bladder was squeezed to assist micturition once a day until the mice were executed.

### Behavioral analysis

The Basso Mouse Scale (BMS) open field test was used to evaluate the motor function of hind limbs in mice. Mice were evaluated once a week before and after injury by the same two observers without awareness of experimental conditions in a double-blinded manner.

### Electrophysiology analysis

Electrophysiological testing was performed for each group by using Keypoint II dual-channel evoked potential/electromyography (Dantech). All animals were anesthetized by intramuscular (IM) injections of ketamine (20 mg/kg). For motor-evoked-potential (MEP) recording, two stimulating electrodes were included the positive electrode was placed on the skull surface of the motor area of the cerebral cortex (AP + 1.0, L/R ± 1.5, DV 0, mm from Bregma), 1 mm behind the bregma and 1.5 mm on the left or right side from the midline; and the negative electrode was placed on the skull 0.5 cm lateral to the positive electrode. The recording electrode was inserted into the left or right gastrocnemius muscle of hind limbs with a depth of 1.5 mm. Moreover, the reference electrode was placed at 2 cm away from the recording electrode and the grounding line was placed in the middle of the stimulating electrode and recording electrode. 0–10 mA single square wave (1 Hz) was applied to stimulate the motor area of the cerebral cortex through the skull with a duration of 0.2 ms. Two features of MEP were recorded at the gastrocnemius muscle of the hindlimb, i.e., peak-to-peak amplitudes were calculated as amplitude values and the onset time of the first response to the stimulus was measured as latency.^53^

### Tissue processing

After excessive inhalation of isoflurane, animals were transcardially perfused with 4% polyformaldehyde (PFA, Sigma) in phosphate buffer saline (PBS, pH 7.4, Sigma). The spinal cord was removed and placed overnight in 4% PFA at 4 °C, then transferred to 30% sucrose (Sigma) twice and placed overnight at 4 °C. Samples were photographed under a microscope (Nikon) then using tissue embedding media, OCT (Thermo Scientific), a 1 cm spinal cord segment centered on the lesion core was encapsulated in OCT on dry ice. Tissue sections were cut at a thickness of 15 μm using a cryostat (Leica) and mounted on charged glass slides. The sections were stained by hematoxylin–eosin (H–E, Sigma) to observe the histological structure of the tissue after SCI.

### Immunohistochemistry

Sections were washed three times with 1× PBS and then incubated with the primary antibodies at 4 °C overnight after 1 h blocking by 5% normal goat serum (NGS, Sigma) and 0.2% Triton X-100 (Sigma). The sections were then incubated at room temperature for 2 h with fluorescent-labeled secondary antibodies (Invitrogen) and washed with 0.01 M PBS 3 times before being observed under a confocal laser scanning microscope (Zeiss, LSM800). Fluorescence immunohistochemistry was performed using the following primary antibodies: rabbit anti-NeuN (Abcam, 1:500), chicken anti-MAP2 (Abcam, 1:500), rabbit anti-GFAP (Dako, 1:1000), chicken anti-GFAP (Abcam, 1:1000), rat anti-CD45 (ebioscience, 1:500), rat anti-CD11b (ebioscience, 1:500), guineapig anti-IBA1 (Synaptic Systems, 1:800), rabbit anti-SLC1A3 (EAAT1/GLAST) (Abcam, 1:500), mouse anti-AQP4 (Abcam, 1:350), rabbit anti-SOX2 (Abcam, 1:500), rabbit anti-MKI67 (Abcam, 1:500).

### Image quantification

For each group of experimental animals (*N* ≥ 3), longitudinal slices containing the central spinal canal were selected. The target areas were photographed by 20× objective lens without optical zoom (Plan-Apochromat, NA = 1) using confocal laser scanning microscopy at a resolution of 1024 × 1024 pixels. Each region was scanned along the *z*-axis at intervals of 1.5–2.5 μm. A final high-definition 3-D image was achieved via reconstructing these consecutive scans using the software Imaris (Bitplane). We then used Imaris to determine cell counts and regions of interest. All figures were composed with Adobe Photoshop, Graphpad Prism, and Adobe Illustrator.

### Western blot analysis

The 1 cm long spinal cord segments centered on the lesion core were dissected and collected from mice at different time points post SCI. Such tissue samples were homogenized in 500 μL RIPA buffer (Beyotime) with 1 mmol/L phenylmethanesulfonyl fluoride and a mixture of protease inhibitor cocktails. After 10 min incubation on ice, they were then centrifuged at 12,000 rpm for 30 min at 4 °C and quantitated using a BCA quantification kit. Twenty-five micrograms (25 μg) of protein of each sample was loaded on 10% acrylamide gels. The antibodies used for Western blotting were rabbit anti-Snap25 antibody (SYSY) and anti-beta actin antibody (Abcam).

### Statistical analysis

Graphpad Prism was used for statistics. The data were expressed as mean (±SEM). Student *t*-test and unpaired Student *t*-test were used to determine the statistical difference between the two groups. *P* < 0.05 was considered to indicate statistically significant differences.

### Bulk-RNA-Seq

The 1 cm long spinal cord segments centered on lesion core, an average of 20 mg, at 15 min, 1 d, 3 d, 7 d, 14 d, 28 d, and 42 d after injury, were homogenized in 1 mL TRIzol (Invitrogen), and mRNA was extracted and purified by RNeasy Mini Kit (QIAGEN) according to the manufacturer’s instructions. The qualities of RNA were determined by Agilent 2100. Totally, 1–3 μg qualified RNA were subjected to Illumina V2 RNAseq library construction, followed by Hiseq 2000 SE50bp Sequencing.

### WGCNA

By first creating a paired correlation matrix between all annotated gene pairs, a signed weighted correlation network was constructed using 39 samples. The resulting Pearson correlation matrix was converted into a connection strength matrix (an adjacency matrix). Topological overlap was then calculated to measure network interconnectivity. For each dataset, we grouped genes for network connection strength based on their topological overlap similarity and difference measure (1 - topological overlap) using average-linkage hierarchical clustering. Using the dynamic tree-cutting algorithm and the threshold of merge function set to 0.25, we identified 7 modules.

### TF network analysis

The differentially expressed genes (DEGs) at each time point were integrated into TF enrichment analysis through the Database for Annotation, Visualization, and Integrated Discovery (DAVID) online tools (version 6.8, https://david.ncifcrf.gov/), respectively.^54^ Furthermore, the co-expression analysis between significantly enriched TFs and DEGs was conducted and only TF-DEG pairs with the absolute value of Spearman correlation coefficient greater than 0.90 were filtered to construct the TF regulatory network at different time points. Eventually, the key TF-DEG pairs were illustrated as the TF regulatory network by the Cytoscape software (version 3.7.0).^55^

### ScRNA-Seq

The 10 mm long spinal cord segments centered on the lesion core were collected at different time points after SCI, dissociated in papain at a concentration of 2 mg/ml in Hibernate A-Ca (HE-Ca, BrainBits, LLC) at 30 °C for 10 min. The digested tissues were gently triturated 10 times with fire-polished Pasteur pipettes. The single-cell suspension was filtered through a 40 μm cell strainer and then centrifuged at 200 g for 5 min at room temperature. The pellet was washed with PBS containing 0.04% bovine serum albumin (BSA). Cell viabilities were assessed by trypan blue staining. The cells were diluted to a final concentration of 1 × 10^6^/mL in PBS with 0.04% BSA. The volume of single-cell suspension that was required to generate 10,000 single-cell GEMs (gel beads in the emulsion) per sample was loaded onto the Chromium Controller (10× Genomics). Libraries were prepared using the Chromium v3 Single Cell 3′ Library and Gel Bead Kit (10× Genomics) according to the manufacturer’s specifications. Final library quantification and quality control were performed using a DNA 1000 chip (Agilent Technologies), followed by sequencing on Illumina NovaSeq 6000.

Raw sequence data were aligned to the mm10 (Ensembl 84) reference genome, and cell numbers along with unique molecular identifiers (UMIs) were estimated, using the CellRanger (version 3.1.0), the single-cell software suite from 10× Genomics. Downstream analyses were performed using the Seurat R package (version 3.1.5). Low-quality cells elimination was performed according to previous strategies.^56^ Cell libraries with low complexity (fewer than 200 expressed genes) were excluded. Cells with mitochondrial gene-expression fractions greater than thresholds for each sample were excluded. The thresholds were determined by considering a median-centered median absolute deviation-variance normal distribution; cells with mitochondrial read fraction outside of the upper end of this distribution were excluded (where outside corresponds to *P* < 0.05; Benjamini–Hochberg-corrected).

### Clustering and cell-type classification

All Seurat objects were combined together. Cells with mitochondrial expression levels below 10% were screened out using feature counts larger than 500 and RNA counts below 5000. The LogNormalize method with a scale factor of 10,000 was used for normalization. The top 5,000 variable features were extracted using the FindVariableFeatures function. The data were scaled according to the mitochondrial percentage using the ScaleData function. The clustering results were visualized using t-SNE and UMAP plots. The function FindAllMarkers was used to find the DEGs in each cluster. Cell types were identified by using several marker genes from the literature.

## Supplementary information

Supplementary Material

## Data Availability

The sequencing datasets that support the findings of this study are available in figshare with the identifier (10.6084/m9.figshare.17702045).
