# Correction to: Temporal and spatial cellular and molecular pathological alterations with single-cell resolution in the adult spinal cord after injury

**DOI:** 10.1038/s41392-022-01012-z

**Published:** 2022-05-10

**Authors:** Chen Li, Zhourui Wu, Liqiang Zhou, Jingliang Shao, Xiao Hu, Wei Xu, Yilong Ren, Xingfei Zhu, Weihong Ge, Kunshan Zhang, Jiping Liu, Runzhi Huang, Jing Yu, Dandan Luo, Xuejiao Yang, Wenmin Zhu, Rongrong Zhu, Changhong Zheng, Yi Eve Sun, Liming Cheng

**Affiliations:** 1 Division of Spine, Department of Orthopaedics, Tongji Hospital, Tongji University School of Medicine, Shanghai, 200065 China; 2 Key Laboratory of Spine and Spinal cord Injury Repair and Regeneration (Tongji University), Ministry of Education, Shanghai, 200072 China; 3 Institute of Spinal and Spinal Cord Injury, Tongji University School of Medicine, Shanghai, 200065 China; 4 Stem Cell Translational Research Center, Tongji Hospital, Tongji University School of Medicine, Shanghai, 200065 China; 5 Department of Psychiatry and Biobehavioral Sciences, David Geffen School of Medicine, University of California Los Angeles, Los Angeles, CA 90095 USA

**Keywords:** Cellular neuroscience, Diseases of the nervous system, Regeneration and repair in the nervous system

Correction to: *Signal Transduction and Targeted Therapy* 10.1038/s41392-022-00885-4, published online 02 March 2022

After online publication of the article^1^, the authors noticed the inadvertent mistakes occurred in Fig. 2g & Fig. 4a that needs to be corrected. The correct data are provided as follows. The key findings of the article are not affected by these corrections. The original article has been corrected.

**Fig. 2:**
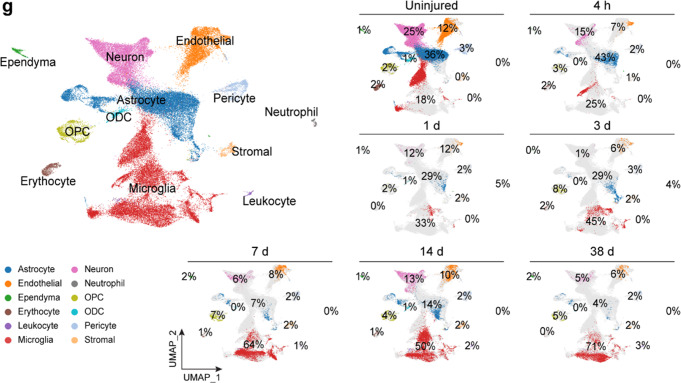
**g** UMAP visualization plot of 59,558 spinal cord cells sequenced from all samples, color-coding defined 12 major cell types based on signature gene expression. The panels on the right show the proportion of each cell type at each time point before and post SCI

**Fig. 4:**
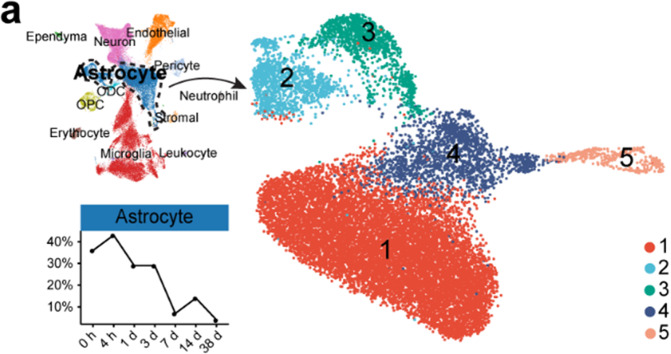
**a** UMAP visualization plot showing 5 astrocyte clusters (subtypes)

The authors mistakenly switched “4h” and “1d” UMAP plots. The correct version of Fig. 2g is shown above.

The authos mistakenly spelled “Leukocyte” as “Leukecyte”. The correct version of Fig. 4a is shown above.
