# Supplementary material for: Temporal and spatial cellular and molecular pathological alterations with single-cell resolution in the adult spinal cord after injury
Source: Signal Transduct Target Ther. 2022 Mar 2;7:65. doi: 10.1038/s41392-022-00885-4 (PMC8888618; doi:10.1038/s41392-022-00885-4)
Supplement: Supplementary file 1 — Supplementary Material [file 41392_2022_885_MOESM1_ESM.docx]

Supplementary Materials for

Temporal and Spatial Cellular and Molecular Pathological Alterations with Single Cell Resolution in Adult Spinal Cord after Injury

Chen Li^1, 2, 3, 4 #^, Zhourui Wu^1, 2, 3, 4 #^, Liqiang Zhou^3, 4 #^, Jingliang Shao^3, 4^ , Xiao Hu^1, 2, 3, 4^, Wei Xu^1, 2, 3, 4^, Yilong Ren^1, 2, 3, 4^, Xingfei Zhu^2, 3, 4^, Weihong Ge^5^, Kunshan Zhang^3, 4^, Jiping Liu^3, 4^, Runzhi Huang^2, 3, 4^, Jing Yu^3, 4^, Dandan Luo^3, 4^, Xuejiao Yang^3, 4^ , Wenmin Zhu^3, 4^, Rongrong Zhu^2, 3, 4^, Changhong Zheng^3, 4^, Yi Eve Sun^2, 3, 4^ *, Liming Cheng^1, 2, 3, 4 *^

Correspondence to: limingcheng@tongji.edu.cn; yi.eve.sun@gmail.com.

**This PDF file includes:**

Figures. S1 to S11

Tables S1


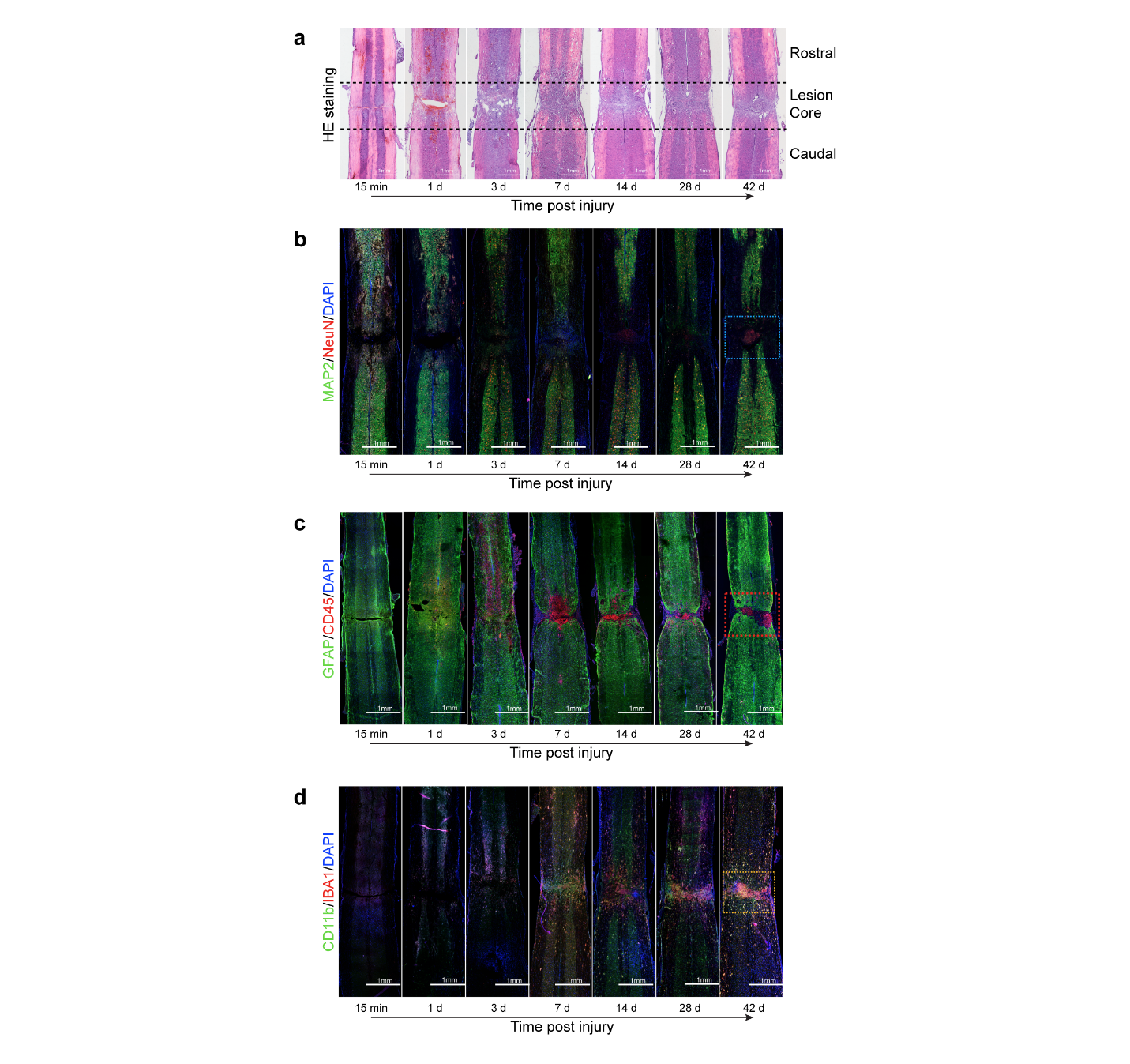


Figure. S1. Temporal changes revealed by histological analysis of mouse spinal cord post SCI.

(**a**) H&E staining showing lesion core changes from 0 d to 42 d post SCI. (**b**) Immunofluorescent staining with neuronal markers MAP2/NeuN from 0 d to 42 d after SCI. (**c**) Immunofluorescent staining with astrocyte marker GFAP and pan-leukocyte marker CD45 from 0 d to 42 d after SCI. (**d**) Immunofluorescent staining with microglia markers IBA1/CD11b from 0 d to 42 d post SCI.


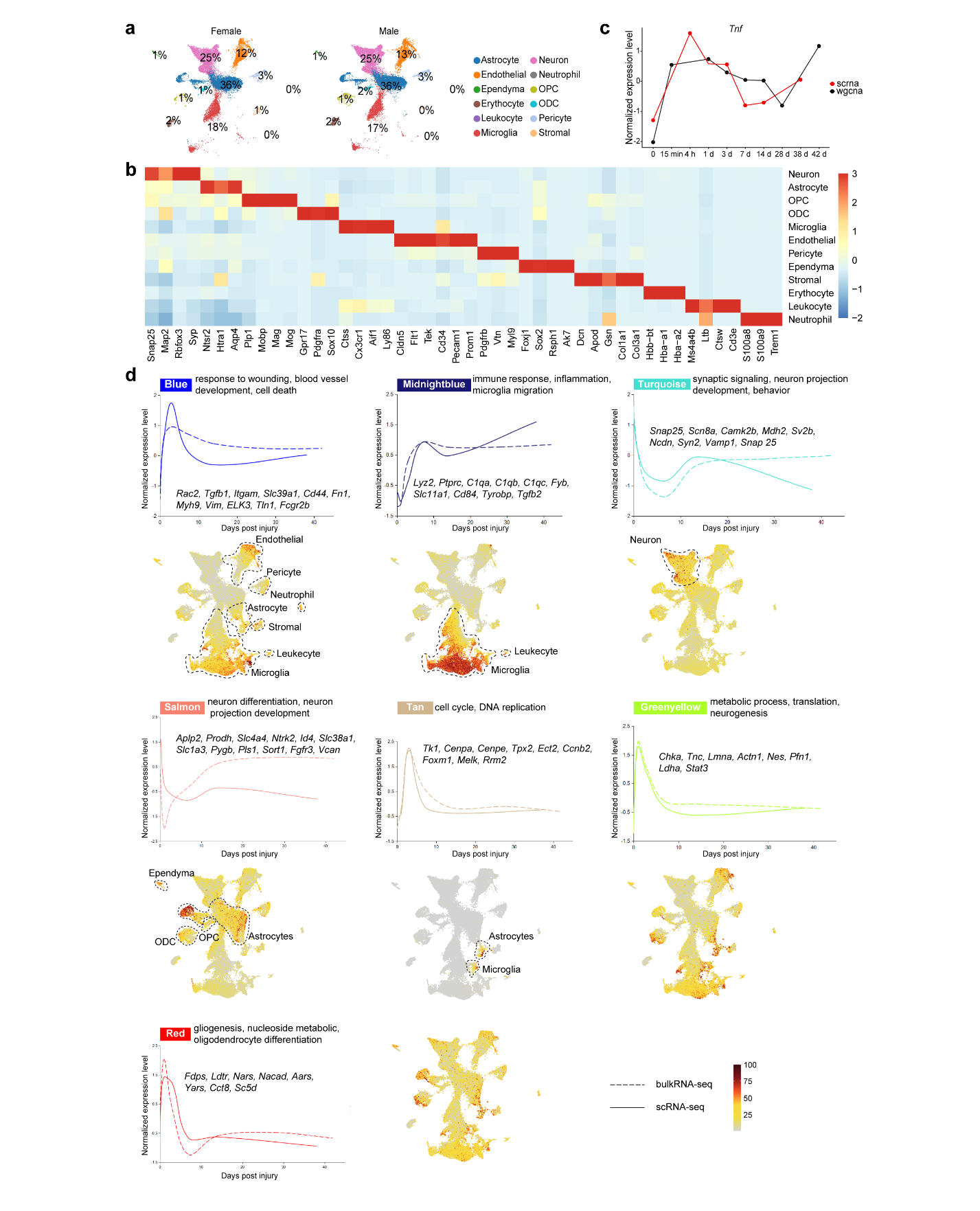


Figure. S2. The population-based and scRNA-seq data demonstrating consistent molecular changes after SCI.

(**a**) UMAP visualization plots show scRNA-seq results between male and female uninjured spinal cord were very similar. (**b**) Heatmap of normalized mean expression for key signature genes for each major cell type. (**c**) Line charts demonstrating temporal changes of gene *Tnf* in bulk-RNA- and scRNA-seq data. Both demonstrated a 2nd wave of increased expression after 14 days post SCI. (**d**) Temporal changes of 7 modules identified by bulk-RNA-seq of spinal cord tissues after SCI, as presented by averaged expression of each module’s top 20 genes, and averaged expression of the same top 20 genes detected from the scRNA-seq data. Result demonstrates good consistency between bulk-RNA-seq and scRNA-seq results. GO terms of each module are also presented. Feature plots showing averaged expression patterns of each module’s top 20 genes in each cell types based on scRNA-seq results. Hub genes of each module were also listed on line charts.


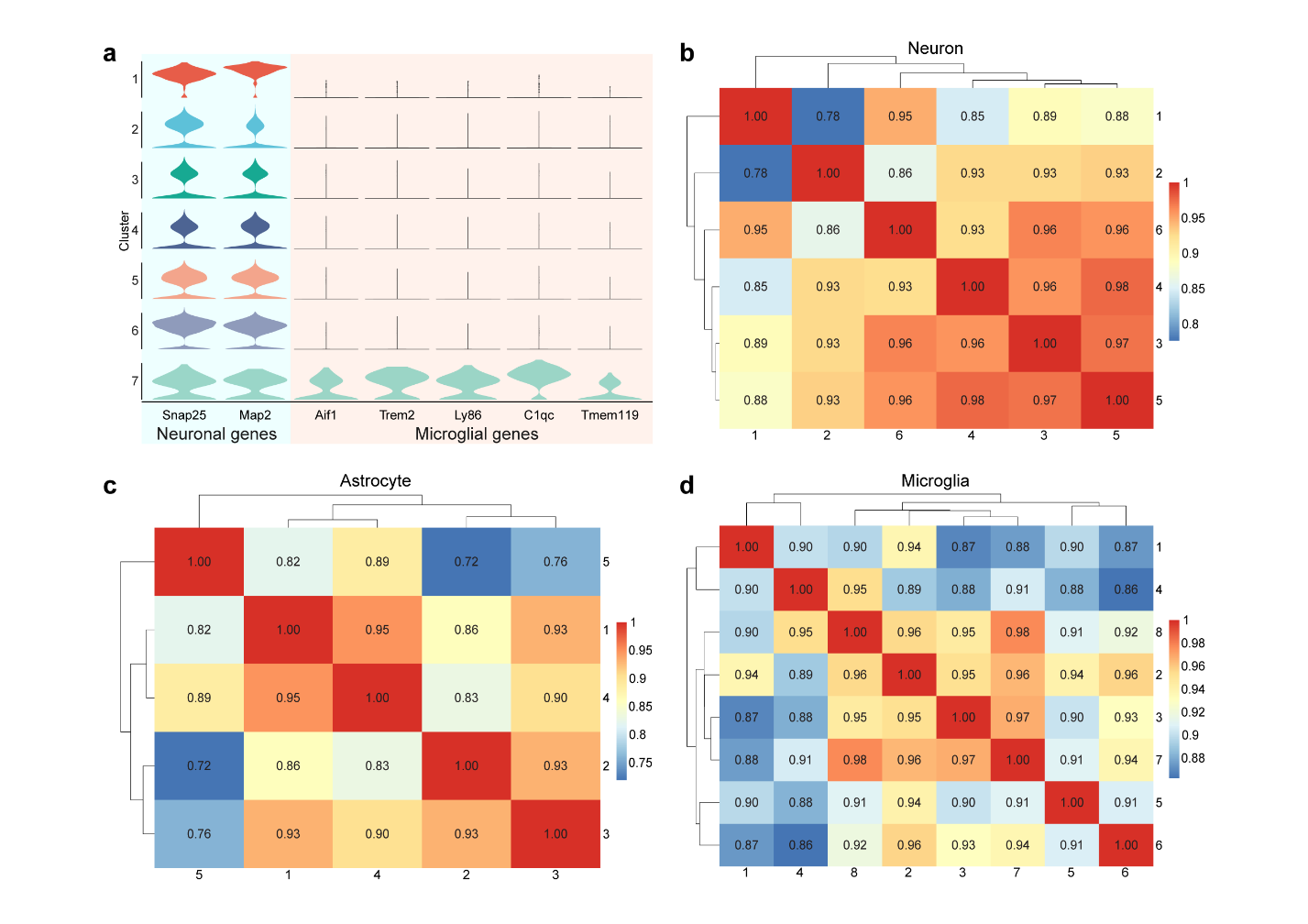


Figure. S3. Relationships between each cluster (subtype) within neuron/astrocyte/microglia-specific populations.

(**a**) Violin plots showing expression of neuronal signature genes and microglial signature genes in each neuronal cluster (subtype). Cluster 7 cells expressed both neuronal and microglial signature genes and therefore might be doublets and were therefore omitted from subsequent analyses. (**b**) Clustering heatmap based on Pearson’s correlation coefficient of transcriptomes between every pair of neuronal clusters (subtypes). (**c**) Clustering heatmap based on Pearson’s correlation coefficient of transcriptomes between every pair of astrocytic clusters (subtypes). (**d**) Clustering heatmap based on Pearson’s correlation coefficient of transcriptomes between every pair of microglial clusters (subtypes).


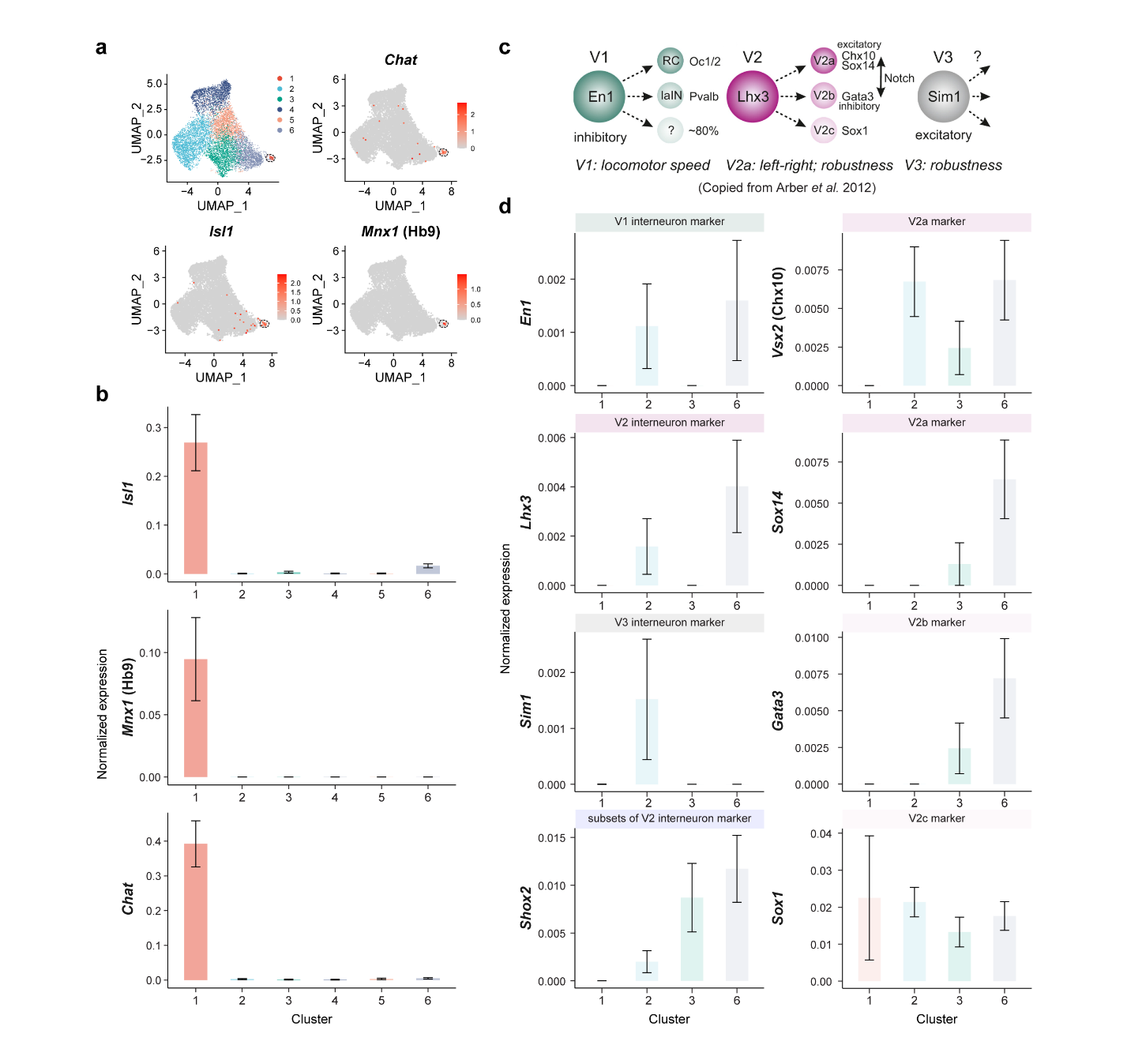


Figure. S4. Expression pattern of typical motor neuron and ventral interneuron marker genes in neuronal population revealed by scRNA-seq.

(**a**) UMAP visualization of typical motor neuron specific genes (*Chat*, *Isl1*, *Mnx1*(HB9)) in neuronal populations. Dashed curve highlights cluster 1. (**b**) Bar plots showing expression of motor neuron specific genes in 6 neuronal subpopulations. (**c**) Subtypes of mouse spinal cord ventral interneurons revealed in Arber (2012). (**d**) Subtypes of mouse spinal cord ventral interneurons revealed in Arber (2012).


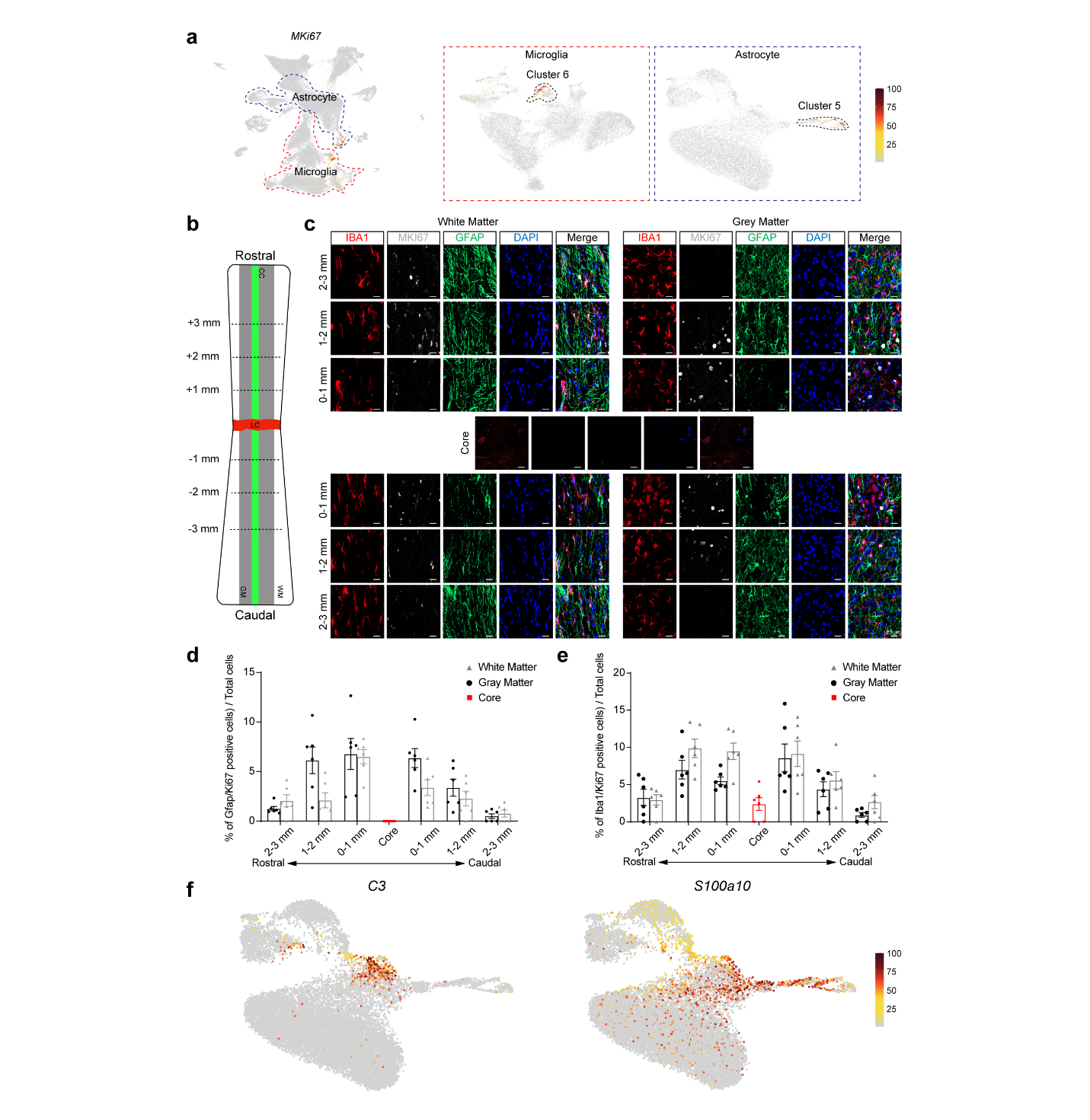


Figure. S5. Proliferations of astrocytes and microglia peaked at 3d post SCI.

(**a**) Feature plots showing gene expression of cell proliferation marker *Mki67* in UMAP template demonstrating subpopulations of astrocytes and microglia represented two major proliferating cell types in the spinal cord after SCI. (**b**) Schematic diagram of regions of interest used for immunofluorescence analyses in **c** and statistical analyses in **d-e**. (**c**) Immunofluorescent staining of different regions (indicated in **b**) of spinal cord tissues with microglia marker IBA1 (*Aif1*), astrocyte marker GFAP and cell proliferation marker MKI67 at 3 d post SCI. (**d-e**) The statistical graph showing the distribution of proliferating cells in spinal cord tissues encompassing the lesion core. Note that proliferating astrocytes were excluded from the lesion core, whereas proliferating microglia could be detected within the lesion core.


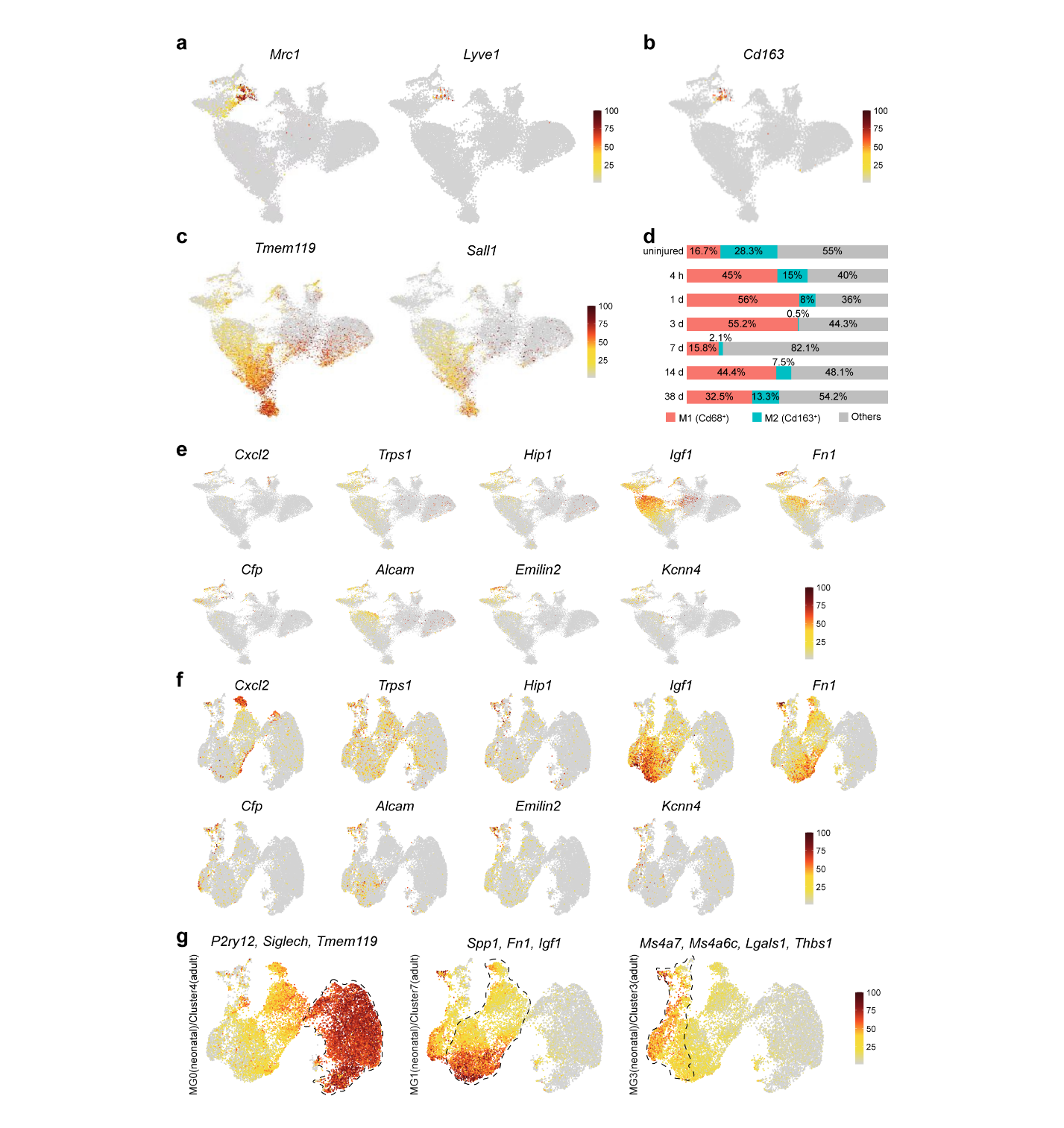


Figure. S6. In depth analyses of microglial population detected by scRNA-seq.

(**a**) Feature plots showing gene expression of *Mrc1* and *Lyve1* in different microglial clusters. *Mrc1* and *Lyve1* had been reported to be macrophage markers. (**b**) Feature plots showing gene expression of M2 marker *Cd163* in different microglial clusters (subtypes). (**c**) Feature plots showing gene expression of classic microglia gene *Tmem119* and *Sall1* in different microglial clusters. These two genes were considered classic microglia signature genes. (**d**) Bar graph showing percentage of putative M1/M2 cells (based on *Cd68* and *Cd163* expression) in microglial cluster 3 at different time points post SCI. (**e**) Feature plots showing gene expression of each repopulating microglia signature genes in different adult spinal cord microglia clusters. (**f**) Feature plots showing gene expression of each repopulation microglia signature genes in MG 0/1/3 (neonatal) and cluster 3/4/7 (adult) in combined scRNA-seq data set. (**g**) Feature plots showing averaged expression of MG 0/1/3-specific signature genes in neonatal microglia MG 0/1/3 and adult spinal cord microglia cluster 3/4/7 combined scRNA-seq data set.


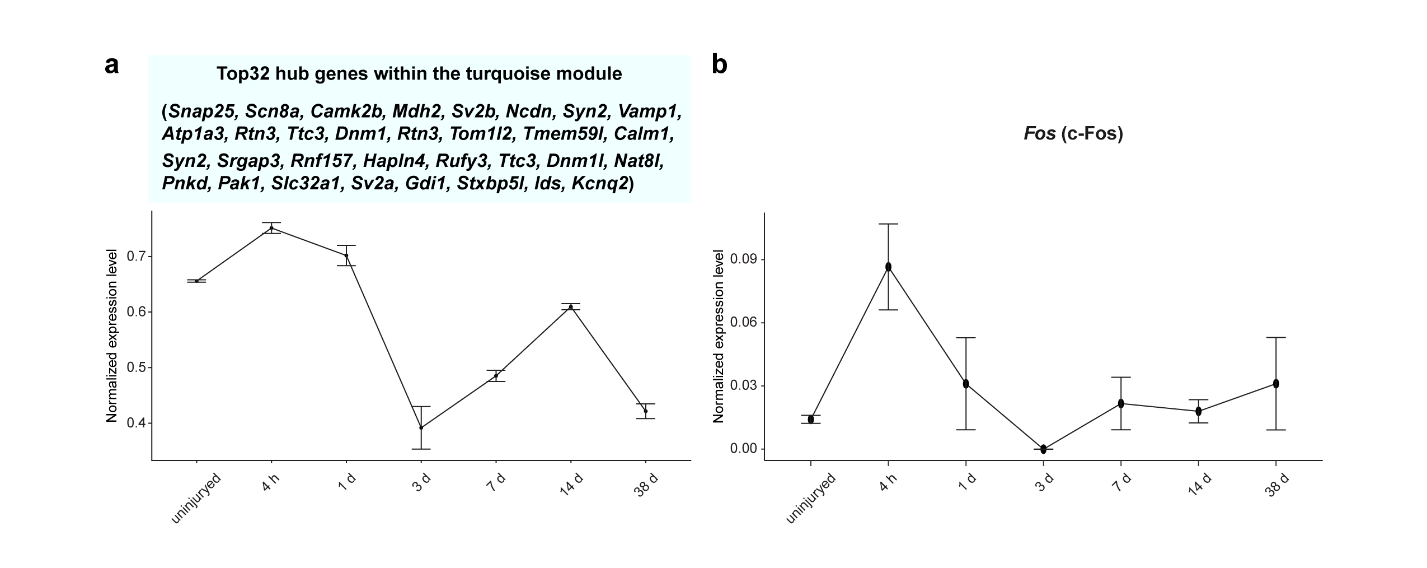


Figure. S7. Time-course of the expression of neurotransmission-related genes and immediate early gene, Fos, in neuronal populations after SCI revealed by scRNA-seq.

(**a**) Time-course of the expression of top32 hub genes within the turquoise module in neuronal populations. (**b**) Time-course of the expression of immediate early gene *Fos* (c-Fos) in neuronal populations.


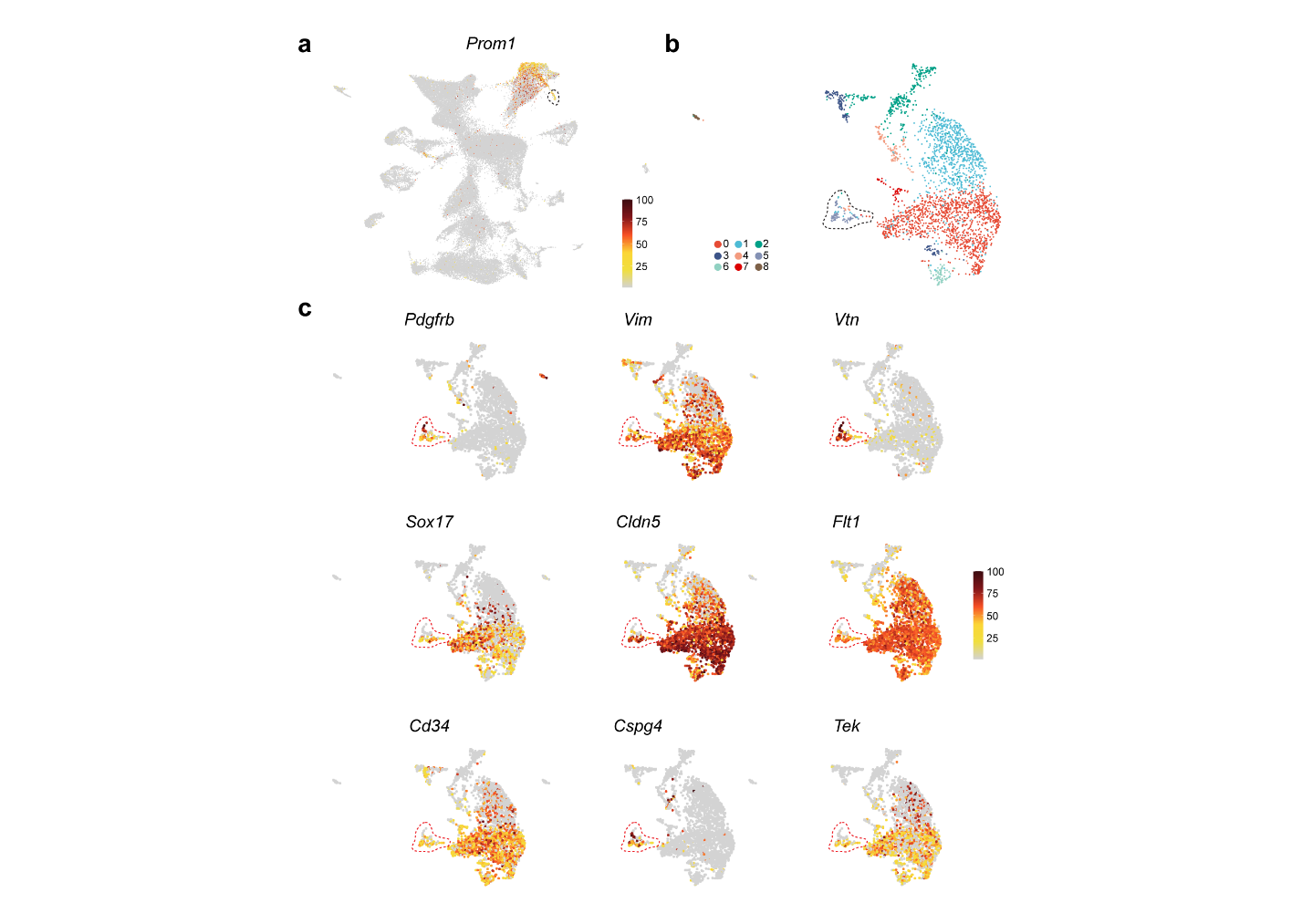


Figure. S8. Defining ependymal CD133+ neural stem cells (NSCs) in adult spinal cord scRNA-seq data.

(**a**) Feature plots showing gene expression of *Prom1* (CD133) in all cell types. (**b**) UMAP visualization plot showing all *Prom1* (CD133) positive cells could be divided into 8 subclusters. (**c**) Feature plots showing gene expression of the 4th ventricle NSCs signature genes in all *Prom1* (CD133) expressing cells from adult spinal cord scRNA-seq data.


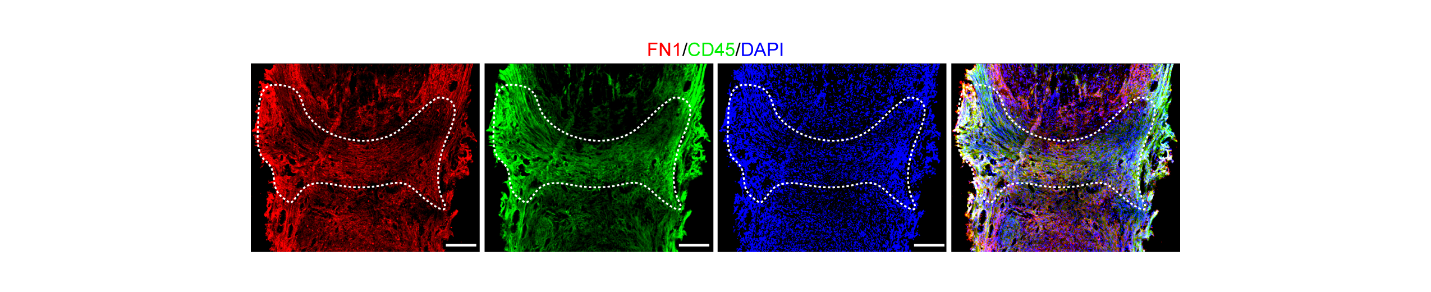


Figure. S9. Immunostaining of FN1 and CD45 in spinal cord 7 days after SCI (Dashed curve showing the boundaries of epicenter region).

**
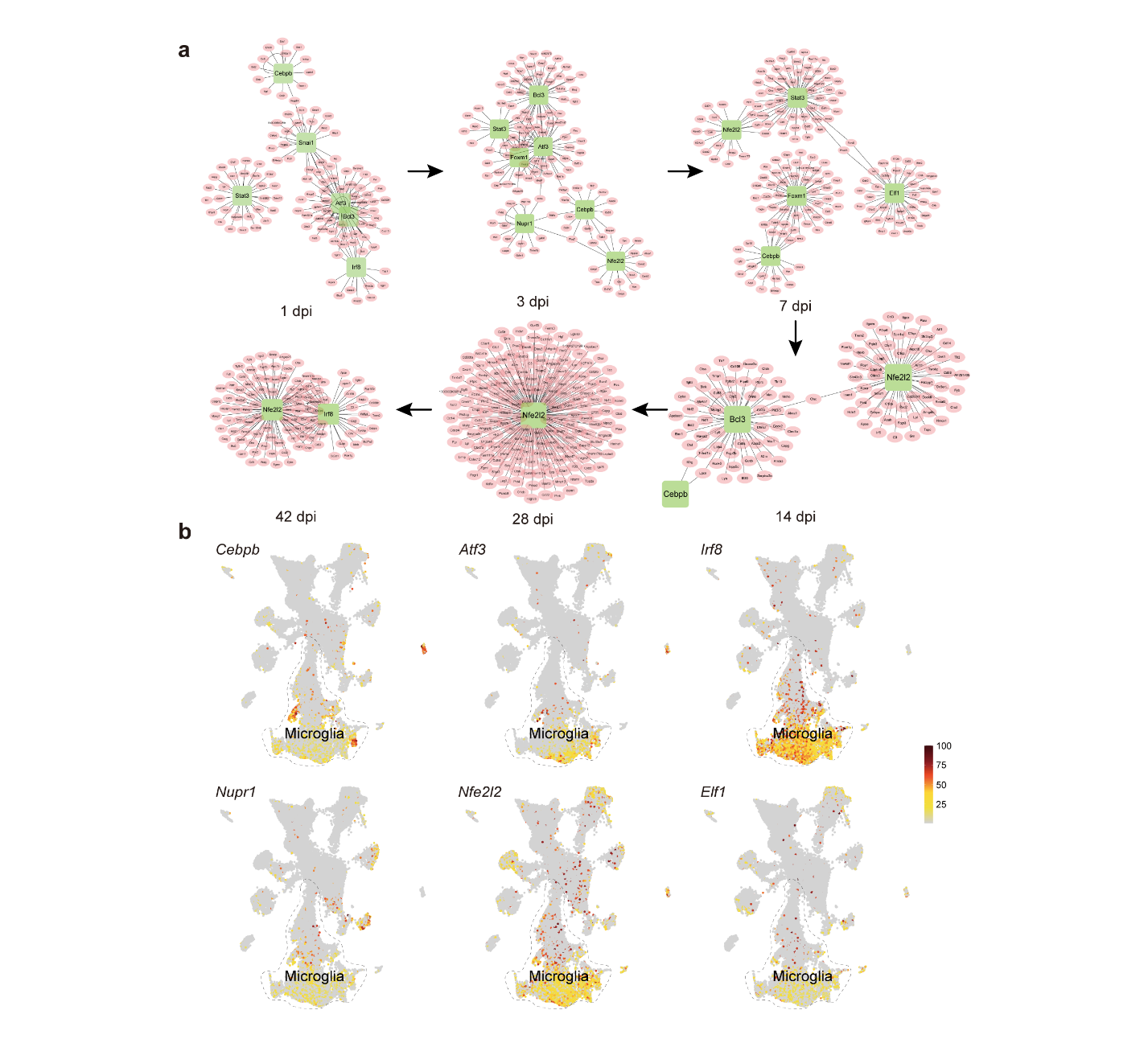
**

Figure. S10. Microglial cells being the featured cells expressing key transcription factors in adult spinal cord after SCI.

(**a**) Transcription factor enrichment analyses of the bulk-sequencing data revealed most significant (key) transcription factors and their target genes expressed at each specific time post SCI. (**b**) Feature plots showing gene expression of major transcription factors in all cell types. Results indicate microglial cells being the major host cells harboring the expression of featured major transcription factors identified from bulk-RNA-seq. dpi, days post injury.


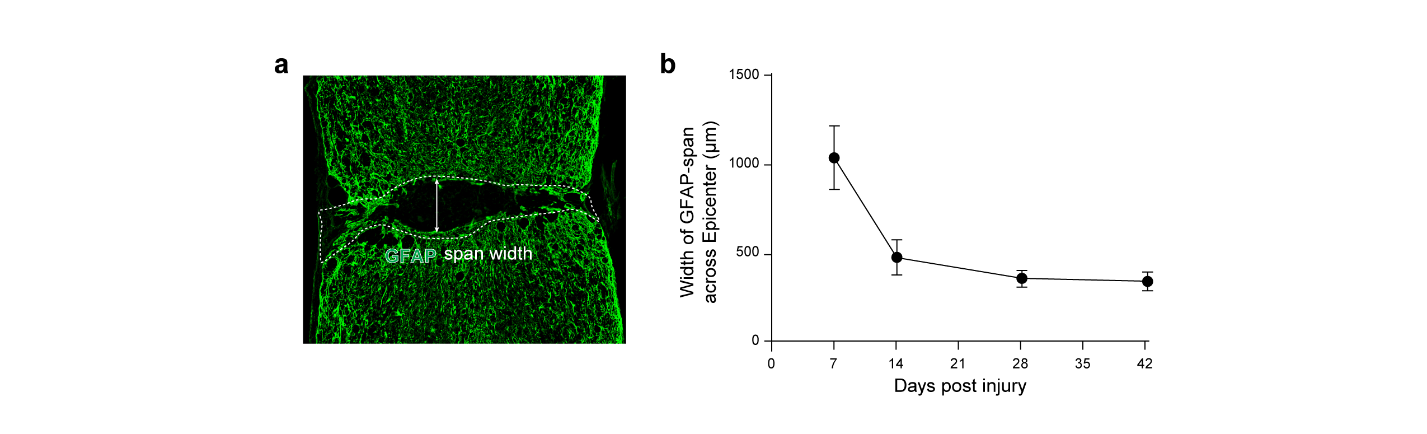


Figure. S11. Lesion stabilization revealed by changes in GFAP span encompassing the epicenter with time after spinal cord injury.

(**a**) An example illustrating the GFAP span across spinal cord epicenter. (**b**) Line chart showing the widths of GFAP-span measured at different times after SCI.

Table S1. Spinal cord injury phases and key pathological events described in previous and this study.

| **Data source and Key Events** | Time Post SCI and Injury Phases | | | | |
| --- | --- | --- | --- | --- | --- |
|  | **0 - 2 h** | **2 h - 2 d** | **2 d - 2 w** | **2 w - 6 M** | **≥ 6 M** |
| **Rowland**  ***et al.* 2008** | immediate | early acute | subacute | intermediate | chronic |
| **Alessandro**  ***et al.* 2015** | immediate | acute | acute | intermediate | chronic |
| **Our current Study** | **0 - 4 h**  **(Phase I)** | **4 h - 3 d**  **(Phase I)** | **3 d - 2 w**  **(Phase II)** | **2 - 7 w**  **(Phase III)** |  |
|  | immediate | early acute | a) late acute  b) early subacute | a) late subacute  b) intermediate |  |
| **Key Events** |  | spinal shock | a) initiation of astroglial scar  b) BBB repair  c) Resolution of edema | lesion stabilization (**Figure S11**) |  |
